# Depletion of Mannose Receptor–Positive Tumor-associated Macrophages via a Peptide-targeted Star-shaped Polyglutamate Inhibits Breast Cancer Progression in Mice

**DOI:** 10.1158/2767-9764.CRC-22-0043

**Published:** 2022-06-28

**Authors:** Anni Lepland, Alessio Malfanti, Uku Haljasorg, Eliana K. Asciutto, Monica Pickholz, Mauro Bringas, Snežana Đorđević, Liis Salumäe, Pärt Peterson, Tambet Teesalu, María J. Vicent, Pablo Scodeller

**Affiliations:** 1 Laboratory of Precision and Nanomedicine, Institute of Biomedicine and Translational Medicine, University of Tartu, Tartu, Estonia.; 2 Polymer Therapeutics Laboratory, Prince Felipe Research Centre, Valencia, Spain.; 3 Molecular Pathology Research Group, Institute of Biomedicine and Translational Medicine, University of Tartu, Tartu, Estonia.; 4 School of Science and Technology, National University of San Martin (UNSAM) ICIFI and CONICET, Buenos Aires, Argentina.; 5 Departamento de Física, Facultad de Ciencias Exactas y Naturales, Universidad de Buenos Aires, Buenos Aires, Argentina.; 6 Instituto de Física de Buenos Aires (IFIBA), CONICET-Universidad de Buenos Aires, Buenos Aires, Argentina.; 7 Departamento de Química Inorgánica, Analítica y Química Física, Facultad de Ciencias Exactas y Naturales, Universidad de Buenos Aires, Buenos Aires, Argentina.; 8 Fundación Instituto Leloir, Instituto de Investigaciones Bioquímicas de Buenos Aires (IIBBA-CONICET), C1405BWE Ciudad Autónoma de Buenos Aires, Buenos Aires, Argentina.; 9 Pathology Department, Tartu University Hospital, Tartu, Estonia.; 10 Centre for Nanomedicine and Department of Cell, Molecular and Developmental Biology, University of California, Santa Barbara, California.; 11 Department of Immunology and Oncology, Centro Nacional de Biotecnología (CNB-CSIC), Madrid, Spain.

## Abstract

Although many studies have explored the depletion of tumor-associated macrophages (TAM) as a therapeutic strategy for solid tumors, currently available compounds suffer from poor efficacy and dose-limiting side effects. Here, we developed a novel TAM-depleting agent (“OximUNO”) that specifically targets CD206^+^ TAMs and demonstrated efficacy in a triple-negative breast cancer (TNBC) mouse model. OximUNO comprises a star-shaped polyglutamate (St-PGA) decorated with the CD206-targeting peptide mUNO that carries the chemotherapeutic drug doxorubicin (DOX). In the TNBC model, a fluorescently labeled mUNO-decorated St-PGA homed to CD206^+^ TAMs within primary lesions and metastases. OximUNO exhibited no acute liver or kidney toxicity *in vivo*. Treatment with OximUNO reduced the progression of primary tumor lesions and pulmonary metastases, significantly diminished the number of CD206^+^ TAMs and increased the CD8/FOXP3 expression ratio (indicating immunomodulation). Our findings suggest the potential benefit of OximUNO as a TAM-depleting agent for TNBC treatment. Importantly, our studies also represent a novel design of a peptide-targeted St-PGA as a targeted therapeutic nanoconjugate.

**Significance:**

A peptide-targeted nanoformulation of DOX exclusively eliminates mannose receptor+ TAMs in breast cancer models, generating response without off-target effects (a drawback of many TAM-depleting agents under clinical study).

## Introduction

Triple-negative breast cancer (TNBC), defined by the lack of the expression of the estrogen receptor, progesterone receptor, and HER2 (1, 2), represents an aggressive breast cancer subtype with poor prognosis (3) that comprises up to 20% of all breast cancer cases (3, 4). Interfering with immune checkpoints signaling [e.g., through the modulation of programmed cell death 1 (PD-1) and its ligand (PD-L1)] represents an alternative treatment strategy for several cancers and is currently being employed in combination with chemotherapy as a neoadjuvant or adjuvant treatment (5–8). The FDA recently granted accelerated approval for a combination of a PD-L1–blocking antibody (atezolizumab, Tecentriq) and nab-paclitaxel (Abraxane; ref. 9) as a first-line treatment for unresectable locally advanced or metastatic TNBC (10). While promising clinical results have resulted, this combinatorial treatment approach suffers from significant obstacles, including the problematic identification and heterogeneity of PD-L1 expression in patients (11), the limited applicability to patients with PD-L1–positive TNBC (only 20%–42% of cases; refs. 12, 13), and the induction of severe side effects (e.g., neutropenia, peripheral neuropathy, and colitis; refs. 10, 14, 15). Other immune checkpoint inhibitors (ICI), including the CTL-associated antigen 4 (CTLA-4) blockers ipilimumab and tremelimumab, are currently under evaluation for TNBC treatment in combination with other drugs (clinical trial identifiers: NCT03606967, NCT02983045); however, anti–CTLA-4 treatments induce severe side effects such as endocrinopathies, myopathy, enterocolitis, and hepatitis (16–19), which narrow their use. Overall, the limited success of alternative treatment options for TNBC has maintained chemotherapy as the standard of care for most patients (20).

The anthracycline drug doxorubicin (DOX), which presents high off-target effects such as cardiotoxicity (21, 22), represents a frequently employed chemotherapeutic for TNBC; however, disease relapse and metastatic development have also been associated with DOX treatment (23). M2 (anti-inflammatory)-polarized tumor-associated macrophages (TAM; ref. 24) found within both primary and metastatic tumor lesions mediate both events (25); furthermore, TAMs represent the main executioners of tumor progression, immunosuppression, and invasion (24–29), and their presence correlates with inadequate therapeutic response and poor prognosis (25). Recent efforts have focused on eliminating TAMs, and several ongoing clinical trials are currently evaluating TAM depletion in combination with treatments such as ICIs (30). The current clinical-stage gold standard for TAM depletion relies on agents that block colony stimulating factor 1 (CSF1) or its receptor CSF1R, such as the small-molecule CSF1R inhibitor PLX3397 (31); however, microglia also expresses CSF1R (32), the inhibition of CSF1R with PLX5622 impacts M1 macrophages (33), and PLX3397 treatment causes edema (34). Clinical data suggest that anti-CSF1R antibodies induce a modest effect (35, 36) and cause severe side effects that include hematologic toxicities (35) and hepatotoxicity by targeting Kupffer cells (35, 36). Overall, these findings highlight the overwhelming need for new TAM depletion strategies.

Notably, both perivascular TAMs associated with disease relapse and therapeutic resistance (24) and metastasis-associated macrophages (37) express the mannose receptor (CD206/MRC1). Perivascular TAMs employ CD206 to navigate the surrounding collagen-dense stroma (38), which favors tumor progression (39, 40).

For the first time, we report the effects of depleting the CD206^+^ subpopulation of TAMs in a metastatic TNBC mouse model through the use of a targeting agent (the mUNO peptide) for a CD206 site different from the mannose-binding site (41–44). Previous studies have employed mannose to target CD206; however, mannose has other receptors besides CD206 (45, 46).

We decorated a three-arm branched biodegradable multivalent polyanion with a defined negative charge and nanometer-size hydrodynamic radius (star-shaped polyglutamate or St-PGA) with mUNO peptide to function as a targeted delivery platform for a chemotherapeutic agent (DOX) conjugated through a bioresponsive linker. St-PGA-DOX-mUNO (referred to as OximUNO) efficiently depleted CD206^+^ TAMs, relieved immunosuppression in the tumor microenvironment (TME) and limited metastasis/tumor growth, thereby supporting OximUNO as an alternative TAM depletion strategy.

Most importantly, this study represents the first described combination of two reported technologies—the St-PGA nanocarrier and the mUNO-targeting peptide. Overall, this OximUNO proof of concept demonstrates the potential of the peptide-targeted St-PGA nanosystem. Our studies lay a foundation for future work using this nanosystem to target other receptors efficiently by changing the targeting peptide.

## Materials and Methods

### Reagents and Solutions

The peptides mUNO (sequence: CSPGAK-COOH) and FAM-mUNO (FAM-Ahx-CSPGAK-COOH) were purchased from TAG Copenhagen and DOX from Sigma-Aldrich. St-PGA was kindly provided by Polypeptide Therapeutic Solution S.L. (PTS). See the Supplementary Data for information on all other reagents and solutions.

Mayer's hematoxylin solution was prepared by dissolving 5 g of aluminium potassium sulphate dodecahydrate (Merck Millipore, catalog no. 1010421000) in 100 mL of water, and adding 1 g of hematoxylin (Merck, catalog no. H9627). After complete dissolution, 0.02 g of sodium iodide (Merck, catalog no. 1065230100) was added and completely dissolved. Then, 2 mL of acetic acid (Sigma-Aldrich, catalog no. 33209) was added, and then the solution was boiled and then cooled. Once ready to use, the solution was filtered using a 0.45-μm filter.

Eosin (5%) solution was prepared by dissolving 0.5 g of Eosin Y (Sigma-Aldrich, catalog no. 230251) in 99 mL water/1 mL acetic acid.

### Cell Culture and Experimental Animals

4T1 cells were purchased from ATCC, and 4T1-GFP cells were a gift from Ruoslahti laboratory (Sanford Burnham Prebys Medical Discovery Institute, La Jolla, CA). 4T1 and 4T1-GFP cells were cultured in RPMI1640 medium (Gibco by Life Technologies, catalog no. 72400-021) supplemented with 10% volume for volume (v/v) FBS (Capricorn Scientific, catalog no. FBS-11A) and 100 IU/mL penicillin/streptomycin (Capricorn Scientific, catalog no. PS-B) at 37°C in the presence of 5% CO_2_. For all animal experiments, 8–12 weeks old female Balb/c mice were used. Animal experiment protocols were approved by the Estonian Ministry of Agriculture (Project #159). All methods were performed in accordance with existing guidelines and regulations.

### Tumor Models

Two tumor models were used for homing studies: the orthotopic TNBC model, where 1 × 10^6^ 4T1 cells in 50 μL of PBS (Lonza, catalog no. 17-512F) were subcutaneously injected into the fourth mammary fat pad, and the experimental metastasis of TNBC model, where 5 × 10^5^ 4T1 cells in 100 μL of PBS were injected intravenously into Balb/c mice.

Two tumor models were used for treatment studies: the orthotopic TNBC model where 5 × 10^4^ 4T1 cells in 50 μL of PBS were injected subcutaneously into fourth mammary fat pad; and the experimental metastasis of TNBC model where 2 × 10^5^ 4T1-GFP cells in 100 μL of PBS were intravenously injected.

### Nanoconjugate Synthesis and Characterization

*In vivo* homing studies used St-PGA-OG (Oregon Green) and St-PGA-OG-mUNO, while *in vitro* cytotoxicity and *in vivo* treatment studies used St-PGA-DOX and St-PGA-DOX-mUNO (“OximUNO”). Detailed synthetic procedures for single nanoconjugates can be found in Supplementary Data.

### Physicochemical Characterization Methods

#### Nuclear Magnetic Resonance Spectroscopy

Nuclear magnetic resonance (NMR) spectra were recorded at 27°C (300 K) on a 300 Ultrashield from Bruker. Data were processed with Mestrenova software. Sample solutions were prepared at the desired concentration in D_2_O or D_2_O supplemented with NaHCO_3_ (0.5 mol/L).

#### UV-visible Analysis

UV-visible (UV-Vis) measurements were performed using JASCO V-630 spectrophotometer at 25°C with 1-cm quartz cells and a spectral bandwidth of 0.5 nm. Spectra analysis was recorded three times in the range of 200–700 nm.

#### Fluorescence Analysis

Fluorescence analysis was performed using a JASCO FP-6500 spectrofluorimeter at 25°C with 1-cm quartz cells.

#### Dynamic Light Scattering

Size measurements were performed using a Malvern ZetasizerNano ZS instrument, supported by a 532 nm laser at a fixed scattering angle of 173°. Nanoconjugate solutions (0.1 mg/mL) were freshly prepared in PBS (10 mmol/L phosphate, 150 mmol/L NaCl), filtered through a 0.45-μm cellulose membrane filter, and measured. Size distribution was measured (diameter, nm) for each polymer in triplicate. Automatic optimization of beam focusing and attenuation was applied for each sample.

#### Zeta Potential Measurements

Zeta potential measurements were performed at 25°C using a Malvern ZetasizerNano ZS instrument, equipped with a 532 nm laser using disposable folded capillary cells, provided by Malvern Instruments Ltd. Nanoconjugate solutions (0.1 mg/mL) were freshly prepared in 1 mmol/L KCl. Solutions were filtered through a 0.45-μm cellulose membrane filter. Zeta potential was measured for each sample per triplicate.

### Molecular Dynamics Simulations

Molecular dynamics (MD) simulations of PGA chains, and mUNO peptide were carried out using the ff19SB force field (47) in the Amber20 MD engine (https://sbgrid.org/software/titles/ambertools). The nanoconjugate system was neutralized using Na^+^ ions and hydrated to account for a total of approximately 920,000 atoms (∼300,000 TIP3P water molecules) in a truncated octahedral box. A hydrogen mass repartitioning strategy was applied on the resulting topology, allowing us a 4 fs integration time step (48). Standard minimization and equilibration protocols were used to reach 300 K and 1 atm., followed by 50 ns of production MD run. The simulations were run under the NVT ensemble [constant number of particles, volume, and temperature through Berendsen thermostat (49)], considering periodic boundary conditions. The SHAKE algorithm was used to fix hydrogen atoms (50). The nonbound cut-off value was set to Angstrom. The central moiety was parameterized using the recommended protocol for the Amber force field. It was necessary to introduce amide bond, angle, and dihedral terms using the ParmEd module to establish the bond of the central molecule to the PGA chains.

### Tumor Homing Studies

Tumors were induced as described in the tumor model section. Tumor homing studies were performed on mice bearing orthotopic TNBC or experimental metastasis of TNBC. Ten days post-induction (p.i) of the orthotopic TNBC or the experimental metastasis of TNBC model, mice were intraperitoneally injected with St-PGA-OG-mUNO (0.41 mg/0.5 mL of PBS) or St-PGA-OG (0.35 mg/0.5 mL of PBS; corresponding to 15 nanomoles of OG, absorbance measured by UV-Vis). The homing of a higher dose of St-PGA-PGA-mUNO (0.82 mg/0.5 mL of PBS) or St-PGA-OG (0.7 mg/0.5 mL of PBS; corresponding to 30 nanomoles of OG) was also analyzed and compared with the homing of FAM-mUNO (30 nanomoles/0.5 mL of PBS). In every case, nanoconjugates or free peptide were circulated for 6 hours, after which time, mice were sacrificed by anesthetic overdose followed by cervical dislocation. Organs and tumors were collected and fixed in cold 4% w/v paraformaldehyde (PFA) in PBS at +4°C for 24 hours, washed in PBS at room temperature for 1 hour and cryoprotected in 15% w/v sucrose (Sigma Life Science, catalog no. S9378) followed by 30% w/v sucrose at 4°C overnight. Cryoprotected and fixed tissues were frozen in optimal cutting temperature (OCT; Leica, catalog no. 14020108926), cryosectioned at 10-μm thickness on Superfrost+ slides (Thermo Fisher Scientific, catalog no. J1800AMNZ) and stored at −20°C. Immunofluorescence staining was performed as described earlier (42). OG was detected using rabbit anti-FITC/Oregon Green (dilution 1/100, Invitrogen by Thermo Fisher Scientific, catalog no. A889) and Alexa Fluor 647 goat anti-rabbit antibody (dilution 1/250, Invitrogen by Thermo Fisher Scientific, catalog no. A21245). CD206 was detected using rat anti-mouse CD206 (dilution 1/150, Bio-Rad, catalog no. MCA2235GA) and Alexa Fluor 546 goat anti-rat antibody (dilution 1/250, Life Technologies, catalog no. A11081). CD86 was detected using rat anti-mouse CD86 (dilution 1/100, BioLegend, catalog no. 105001) and Alexa Fluor 546 goat anti-rat secondary antibody (dilution 1/250). CD11c was detected using hamster anti-mouse CD11c antibody (dilution 1/75, BioLegend, catalog no. 117301) and Alexa Fluor 546 goat anti-hamster secondary antibody (dilution 1/200, Life Technologies, catalog no. A21111). CD31 was detected with rat anti-mouse CD31 (dilution 1/100, BD Biosciences, catalog no. 553370) as primary antibody and with Alexa Fluor 546 goat anti-rat (dilution 1/200, Invitrogen, catalog no. A11081) as secondary antibody. Slides were counterstained using 4′,6-diamidino-2-phenylindole (DAPI, 1 μg/mL in PBS, Sigma-Aldrich, catalog no. D9542-5MG). Coverslips were mounted using mounting medium (Fluoromount-G Electron Microscopy Sciences, catalog no. 17984-25), and sections were imaged using Zeiss confocal microscope (Zeiss LSM-710) and 20× objective. The colocalization analysis between the FAM or OG channel and the CD206 channel was carried out using the “Coloc2” plugin in the Fiji program and selecting the “Pearson *R* value (no threshold)” coefficient. The colocalization values were obtained from at least three representative images per mouse per group and their average and SE were plotted. The OG/FAM mean signal per CD206^+^ cell analysis was measured using ImageJ, taking the mean OG/FAM signal, and dividing it with the number of CD206^+^ cells. Average values were obtained from four images per mouse. *N* = 3 for orthotopic TNBC and *N* = 2 for the homing in experimental metastasis of TNBC.

### Analysis of Tumor and Liver Leakiness

Endogenous IgG immunostaining of orthotopic 4T1 tumors and livers was performed following the same Immunofluorescence (IF) protocol as described above to assess leakiness. Endogenous IgG was detected using Alexa Fluor 647 goat anti-mouse antibody (dilution 1/200, Invitrogen by Thermo Fisher Scientific, catalog no. A21235) and slides were counterstained with DAPI (1 μg/mL in PBS). The coverslips were mounted, and sections were imaged using Zeiss confocal microscope and 20× objective (*N* = 3 tumors).

### PDL1 Expression Analysis in Orthotopic TNBC Tumors

The assessment of PDL1 expression in orthotopic 4T1 tumors followed the IF protocol described above. PDL1 was detected using rat anti-mouse PDL1 (dilution 1/100, BioLegend, catalog no. 124302) as primary antibody and Alexa Fluor 647 goat anti-rat (dilution 1/200, Invitrogen, catalog no. A21247) as the secondary antibody. Slides were counterstained with DAPI (1 μg/mL in PBS), mounted, and imaged using a Zeiss confocal microscope.

### Tumor Homing of Anti-PDL1 in Orthotopic TNBC Tumors

For the homing analysis with anti-PDL1, we injected 1 × 10^6^ 4T1 cells in 50 μL of PBS subcutaneously and 10 days p.i., PD-L1 antibody (5 mg/kg, rat anti-mouse, BioXcell, catalog no. BE0101) was injected intravenously, circulated for 24 hours after which time, mice were sacrificed, organs collected and fixed with PFA. Ten-micron–thick tissue sections were stained with Alexa Fluor 647 goat anti-rat antibody (dilution 1/200), counterstained with DAPI (1 μg/mL in PBS), mounted, and imaged with a Zeiss confocal microscope.

### Plasma Half-life Evaluation for St-PGA-OG-mUNO

Plasma half-life studies were performed as described previously (42). Briefly, healthy female Balb/c mice (*N* = 3) were intraperitoneally injected with St-PGA-OG-mUNO (0.41 mg/0.5 mL of PBS, corresponding to 15 nanomoles OG). Ten microliters of blood was sampled at different timepoints (0, 5, 10, 15, 30, 60, 180, 360, and 1,440 minutes) and mixed with 50 μL of PBS-Heparin solution. Blood samples were centrifuged to obtain plasma (300 × *g* for 5 minutes at room temperature) and OG fluorescence was read with a plate reader (FlexStation II Molecular Devices) at 480 nm excitation/520 nm emission.

### DOX Release Studies

LC/MS was implemented to determine free drug levels, stability, and drug release with OximUNO. The LC/MS system consisted of an ExionLC LC system and AB Sciex QTRAP 4500, a triple quadrupole ion trap hybrid equipped with a Turbo VTM electrospray ionization source. DOX was detected with an internal standard method: 1 μg/mL of daunorubicin (DAU) was used as internal standard, where three calibration curves (in a range from 0.5 to 50 μg/mL DOX) were prepared and used for accurate analysis of DOX in the samples. Both DOX and DAU were detected with positive electrospray ionization mode by following two mass transitions (544.2 m/z → 397 m/z and 544.2 m/z → 379 m/z for DOX, and 528 m/z → 363.1 m/z and 528 m/z → 321.3 m/z for DAU). The obtained LC/MS optimal conditions were as follows: flow rate 0.5 mL/minute; mobile phase − 0.05% trifluoroacetic acid with 70% of acetonitrile; LiChrospher 100 C18 column (125 × 4.0 mm; Merck); column temperature 40°C, 10 μL injection volume.

#### Stability Study of OximUNO Conjugate in PBS, pH 7.4

OximUNO was incubated in 10 mmol/L dPBS (Dulbecco's phosphate-buffered saline) at 37°C at the concentration of 3 mg/mL and with 3 μg/mL of DAU. A total of 100 μL aliquots were collected at defined timepoints (0, 1, 2, 5, 24, 48, 72 hours), extracted with 3 × 250 μL chloroform, and mixed by vortexing for 5 minutes. Organic phases from all three chloroform extracts were collected in one tube, evaporated using speed vacuum, and stored at −20°C. On the day of analysis, dried samples were reconstituted in 300 μL of methanol (LC/MS grade), vortexed for 5 minutes, and centrifuged for 5 minutes at 30,437 × *g*. Supernatants were filtered through a 0.45-μm filter and subjected to LC/MS analysis.

#### Stability Study of OximUNO in the Intraperitoneal Fluid

Intraperitoneal fluid was collected from healthy 8–12 weeks old Balb/c female mice as performed in ref. 51 by collecting the supernatant and discarding the pellet after the centrifugation step. A working solution containing 3 mg/mL of OximUNO and 3 μg/mL of DAU in intraperitoneal fluid was incubated at 37°C. A total of 50 μL aliquots were collected at scheduled timepoints (0, 2, 5, 7, and 24 hours). Samples were then diluted with 100 μL of methanol, sonicated to dissolve DOX, and injected into the LC/MS after filtration through a 0.45-μm filter.

#### Cathepsin B Release Kinetic Studies

Cathepsin B (5 IU) was activated in 2 mmol/L EDTA (Ethylenediaminetetraacetic acid), 5 mmol/L DTT (Dithiothreitol), and 20 mmol/L CH_3_COONa buffer and incubated at 37°C for 15 minutes. In a separate tube, a solution containing 3 mg/mL OximUNO and 3 μg/mL of DAU was prepared with 20 mmol/L CH_3_COONa and incubated at 37°C for 15 minutes. The two solutions were then combined to produce a reaction solution that was incubated at 37°C. A total of 100 μL aliquots were collected at scheduled timepoints (0, 1, 2, 5, 8, 24, 48, 72 hours), and after the addition of 900 μL of dPBS (to adjust the pH level to 7.4), free DOX and DAU were extracted with 2.5 mL of CHCl_3_ three times. Samples were processed as described under “Stability Study of OximUNO conjugate in PBS, pH 7.4”. After CHCl_3_ evaporation, samples were reconstituted with 300 μL of methanol, filtered through a 0.45-μm filter, and subjected to LC/MS analysis. A blank solution was prepared with the same components as the sample solution but without cathepsin B and used as a control sample.

### *In Vitro* Cytotoxicity Assay

Human peripheral blood mononuclear cells (PBMC) were purified from human blood buffy coat using Ficoll Paque Plus (GE Healthcare, catalog no. 17-1440-02) reagent and CD14^+^ microbeads (MACS Miltenyi Biotec, catalog no. 130-050-201) as described previously (42). A total of 1.2 × 10^5^ cells in 50 μL of RPMI1640 medium were seeded on an FBS-coated 96-well plate. To obtain optimal macrophage attachment and M2 resembling, 50 μL of IL4 (50 ng/mL, BioLegend, catalog no. 574002) and MCSF (50 ng/mL, BioLegend, catalog no. 574802) mixture was added to the wells. The medium was replenished by substituting half of the medium with fresh medium containing IL4 and MCSF every other day for 6 days. To obtain M1-resembling macrophages, monocytes were incubated with M-CSF (50 ng/mL) for 6 days, replenishing every other day with fresh medium containing MCSF and on day 6, 50 μL of MCSF, lipopolysaccharide (LPS, 100 ng/mL, Sigma-Aldrich, catalog no. L4391) and IFNγ (20 ng/mL, BioLegend, catalog no. 570202) was added and incubated overnight. On day 7, cells were incubated for 15 minutes at 37°C with OximUNO, St-PGA-DOX, DOX in medium, or free medium as a control (*N* = 3 wells/group). Concentrations used were calculated on the basis of DOX: 33 and 100 μmol/L. (Of note, the dose of OximUNO used for the 33 μmol/L DOX *in vitro* experiments corresponds to the same dose of OximUNO used for both *in vivo* treatment studies). *In vivo*, all treated groups received injections containing 2 mg/kg of DOX, which, assuming the dilution in mouse blood, corresponds to a DOX concentration of 33 μmol/L). After incubation, wells were washed, fresh medium added, and cells incubated for 48 hours at 37°C. After 48 hours, 10 μL of 3-(4,5-dimethylthiazol-2-yl)-2,5-diphenyltetrazolium bromide (MTT, concentration 5 mg/mL, Invitrogen, catalog no. M6494) in PBS was added to each well containing culture medium and incubated for 2.5 hours at 37°C. Medium containing MTT was then removed without removing formed crystals, and 100 μL of isopropanol was added to each well to dissolve crystals. Absorbance was read at 580 nm using a plate reader (Tecan Sunrise) and the corresponding Magellan 7 program. To analyze the CD206 expression of M2-resembling and M1-resembling macrophages, cells were lifted from 24-well plate using cell scraper, washed 2× with full RPMI and once with PBS, seeded on a 96-well plate with conical bottom at a concentration of 1 × 10^5^ cells in 100 μL of RB [“running buffer”: 4 mL 0.5 mol/L EDTA, 100 mL 5% (w/v) BSA in 1 L of PBS] per well, blocked with 0.5 μL of human TruStain FcX (BioLegend, 422302) in 100 μL of RB at +4°C for 30 minutes after which cells were washed with 100 μL of RB and incubated with 0.5 μL of APC anti-human CD206 (BioLegend, 321109) in 100 μL of RB containing 0.25 μL of human TruStain FcX for 25–45 minutes at +4°C in the dark. After that, cells were washed 2× with 200 μL of RB and read using BD Accuri 6 plus (BD Biosciences). As an isotype control, APC mouse IgG (BioLegend, catalog no. 400119) was used. For washing, plate was centrifuged at 350 × *g* for 7 minutes at +4°C.

### *In Vivo* Liver and Kidney Toxicology Studies with OximUNO

Three healthy 12-week-old female Balb/c mice were intraperitoneally injected once with OximUNO (0.704 mg/0.5 mL PBS or 1.408 mg/0.5 mL) and circulated for 48 hours. Then, mice were anesthetized, and blood collected through retro-orbital bleeding into Lithium Heparin tubes (BD Vacutainer, catalog no. 368494). Blood samples were centrifuged at 1,800 × *g* for 15 minutes at +4°C and 400 μL of plasma was collected for analysis. Samples were analyzed in Tartu University Hospital using a Cobas 6000 IT-MW (Roche Diagnostics Gmbh) machine and reagents for creatinine (CREP2, catalog no. 03263991) and alanine aminotransferase (ALTLP, catalog no. 04467388).

For histologic analysis of livers and kidneys, after sacrificing animals, tissues were frozen into block, sectioned at 10-μm thickness and kept at room temperature for approximately 30 minutes before fixing them with ice-cold methanol for 2 minutes at room temperature followed by hematoxylin and eosin (H&E) staining as described under “H&E staining on PFA-fixed cryosections.” Slides were scanned using Leica DM6 B microscope and Leica Aperio Versa 8 slides scanner with 20× zoom and images were analyzed using the ImageScope (version 12.3.3). Slides were then analyzed by pathologists.

To analyze IFNγ in tissues after OximUNO injection, after sacrificing, tissues were fixed with 4% PFA and cryoprotected. Ten-micron–thick tissue sections were stained with rat anti-mouse IFNγ (dilution 1/50, BioLegend, catalog no. 505701) and Alexa Fluor 647 goat anti-rat antibody (dilution 1/200), counterstained with DAPI (1 μg/mL in PBS), mounted, and imaged with a Zeiss confocal microscope and 10× objective.

### OximUNO Treatment of Orthotopic TNBC

A total of 5 × 10^4^ 4T1 cells in 50 μL of PBS were subcutaneously injected into the fourth mammary fat pad of 8–12 weeks old female Balb/c mice. On day 7, mice were sorted into four groups by tumor volume measured using a digital caliper (Mitutoyo). Tumor volume was calculated on the basis of the formula (*W*^2^ × *L*)/2, where *W* is the tumor's width and *L* is the tumor's length. The starting volume for each group was approximately 25 mm^3^, and the number of mice in each group was five. The first intraperitoneal injection of compounds was carried out on day 7, followed by an intraperitoneal injection every other day; nine injections were performed in total. The dose of nanoconjugates was calculated on the basis of DOX, 2 mg/kg per injection (DOX: 39.5 μg/0.5 mL PBS; St-PGA-DOX: 476 μg/0.5 mL PBS; OximUNO: 341 μg/0.5 mL PBS) giving a cumulative dose of DOX of 18 mg/kg. Mouse bodyweight and tumor volumes were monitored every other day. The final injection was on day 25 and all mice were sacrificed on day 28. Tumor tissues were processed as described under “*In vivo* biodistribution studies,” and the lungs and hearts were embedded in paraffin and processed for H&E staining (described below). Tumors were immunostained as described above. CD206 was detected using rat anti-mouse CD206 (dilution 1/200), CD8 using rat anti-mouse CD8 (dilution 1/75 BioLegend, catalog no. 100701), FOXP3 using rat anti-mouse FOXP3 (dilution 1/75, BioLegend, catalog no. 126401) as primary antibodies, Alexa Fluor goat anti-rat 647 (dilution 1/300 for CD206 and 1/200 for CD8, FOXP3,) was used as a secondary antibody for all markers. Slides were counterstained with DAPI (1 μg/mL in PBS) and imaged using a Zeiss confocal microscope with a 10× objective. All five tumors from each group were included in the IF analysis and at least three images per mouse per group were included. Fluorescent signal intensity was calculated using the ImageJ; to account for different amounts of tissue in the different images, only the area containing tissue was selected and the “mean signal intensity” given by the program taken (total integrated intensity divided by the selected area). For this analysis, at least three images per tumor were included.

### Survival Analysis Following OximUNO Treatment of Orthotopic TNBC

For survival analysis, treatment was performed the same way as described above, with *N* = 5 mice in each group. Mice were sacrificed when their tumors reached 1,500 mm^3^. Survival was analyzed using GraphPad Prism (version 9.3.1) to plot Kaplan–Meier survival curves and to perform Mantel–Cox test for statistical analysis.

### H&E Staining in Paraffin-embedded Formalin-fixed Tissues

For H&E staining, 2-μm–thick sections were cut from paraffin-embedded blocks. Slides were warmed at 60°C for 2 minutes before deparaffinizing using xylene (3 × 2 minutes, 1 × 1 minute) followed by 100% ethanol washes (3 × 1 minute), 80% ethanol wash (1 × 1 minute) followed by 1-minute wash in water. Slides were first incubated with ST-1 HemaLast for 30 seconds, followed by ST-2 hematoxylin for 5 minutes after which time, slides were washed in water for 2 minutes. Then, ST-3 differentiator was added for 45 seconds, and slides were washed in water for 1 minute. Next, ST-4 Bluing Agent was added (1 minute), washed for 1 minute in water followed by 1-minute incubation in 80% ethanol, after which time, ST-5 eosin was added and incubated for 1 minute. For rehydration, incubations in 100% ethanol (2 × 30 seconds, 1 × 2 minutes) were carried out and finished with incubations in xylene (2 × 2 minutes). All washes were carried out in tap water. H&E staining was performed in Tartu University Hospital by pathologists using Leica staining automat and ST Infinity H&E Staining System (Leica, catalog no. 38016998). Stained lung sections were scanned using a slide scanner (Leica SCN400) and 20× zoom. Images were analyzed using the QuPath program (version 0.1.2; ref. 52). Five levels approximately 1 mm apart were used for each mouse to obtain comprehensive pulmonary metastases profile. Stained heart sections were also scanned using a slide scanner and analysed with the QuPath program. Tartu University Hospital pathologists assessed cardiotoxicity in hearts and pulmonary metastases.

### Analysis of CD31 Expression and Blood Vessel Count

CD31 expression after treating orthotopic TNBC tumors with OximUNO, St-PGA-DOX, or DOX was detected using rat anti-mouse CD31 (dilution 1/100) and Alexa Fluor 546 goat anti-rat (dilution 1/200) was used as the secondary antibody. Slides were counterstained with DAPI (1 μg/mL in PBS) and imaged using Zeiss confocal microscope with a 10× objective. CD31 expression was calculated using ImageJ and mean signal per field as described under “OximUNO therapy in orthotopic TNBC,” including at least five images per mouse per group, *N* = 5 mice per group. The blood vessel count was calculated from the same images using ImageJ as follows: the image was changed to an 8-bit image, threshold (Triangle algorithm with modifications to account for as much actual CD31 signal as possible) was added, and particles analyzed. At least three images per mouse per group were included in the analysis, *N* = 5 mice per group. Field size was 1.42 mm × 1.42 mm for all images.

### OximUNO Treatment of Experimental Metastasis of TNBC

A total of 2 × 10^5^ 4T1 cells in 100 μL of PBS were intravenously injected into the tail vein of 8–12 week old female Balb/c mice. Treatment with OximUNO, St-PGA-DOX, or DOX began on day 4 p.i.; each group comprised 6 mice. Doses of different compounds were calculated on the basis of DOX (2 mg/kg): DOX: 39.5 μg/0.5 mL PBS; St-PGA-DOX: 774.5 μg/0.5 mL PBS; OximUNO: 704 μg/0.5 mL PBS. Mouse bodyweight was monitored every other day. A total of six injections were carried out every other day. The final injection was on day 12, and all animals were sacrificed on day 18 using anesthetic overdose and perfusion with PBS. Three right lungs from each group were analyzed with flow cytometry (FC), and three full lungs and three left lungs from each group were frozen into blocks using OCT. Frozen lung tissues were cryosectioned as described earlier, fixed for 10 minutes with cold 4% PFA (CD206) or acetone (for CD8 and FOXP3), and stained as described in the following section. Immunofluorescence staining was performed using the same markers and antibodies as shown in the “OximUNO treatment in orthotopic TNBC” section.

### GFP Staining and Imaging

Six lungs from each group were frozen in OCT. Ten-micron–thick sections were cut and slides were kept at −20°C until ready to use. Slides were taken out of the freezer at least 30 minutes before staining. For staining, slides were fixed with 4% PFA for 10 minutes at room temperature, washed with PBS for 10 minutes at room temperature, counterstained using DAPI (1 μg/mL in PBS) for 5 minutes at room temperature, washed 3 × 4 minutes with PBS and finally mounted using mounting medium. Permeabilization was not used in this step to improve GFP visualization. GFP was visualized using its native fluorescence. Slides were imaged using Olympus confocal microscope (FV1200MPE) with a 10× objective.

### Macroscopic Analysis of GFP Signal

Lungs from each group were imaged using Illumatool Bright Light System LT-9900 (LightTool's Research) in the green channel to visualize the fluorescent signal macroscopically, and a photograph of each lung was taken. The total GFP signal of each lung was quantified by ImageJ using the “IntDen” value.

### FC Analysis

Three mice were sacrificed using anesthetic overdose, perfused with PBS and right lung tissues were placed in cold RPMI1640 medium supplemented with 2% v/v FBS. Lungs were cut into small pieces on ice in a solution containing collagenase IV (160 U/mL, Gibco, catalog no.17104019)/dispase (0.6 U/mL, Gibco, catalog no. 17105-041)/DNase I (15 U/mL; AppliChem, catalog no. A3778) mixture. To obtain a single-cell suspension, lung pieces were incubated in 10 mL of the same mixture at 37°C on a rotating platform for 45–60 minutes, pipetting every 10 minutes to improve digestion. The cells were washed with 5 mL of RB, centrifuged (350 × *g*, 7 minutes, 4°C), and red blood cells were lysed with 3 mL of ammonium-chloride-potassium lysing buffer at room temperature. A total of 10 mL of RB was added; cells were centrifuged and filtered using a 100-μm cell strainer (Falcon, catalog no. 352360). Cells were counted using the bright-field mode of LUNA Automated Cell counter (Logos Biosystems). Cells were collected in RB at a concentration of 5 × 10^6^/100 μL, placed on a 96-well plate with conical bottom and incubated for 30 minutes in FcR-blocking 2.4G2 hybridoma medium at 4°C. The cells were then stained for either macrophage or T-cell markers for 25–45 minutes in the dark at +4°C, centrifuged and washed twice with RB. The antibodies used are listed in Table 1. For intracellular staining of T cells, cells were fixed using eBioscience FOXP3/Transcription Factor Staining Buffer Set (Thermo Fisher Scientific, catalog no. 00-5523-00) according to the protocol provided. Cells were stained for 25–45 minutes in the dark at room temperature following permeabilization and washed twice using RB. All cells were collected in 150 μL of RB, filtered through a 70-μm filter (Share Group Limited) and 150 μL of RB was used to wash the filter. BD LSRFortessa Flow Cytometer and FCS Express 7 Flow (De Novo Software) were used for analysis.

**TABLE 1:** Antibodies used in FC analysis: macrophage and T-cell markers

	Antibody	Dilution, Company
Macrophage markers	PerCP/Cyanine5.5 anti-mouse CD206 (MMR)	1/200, BioLegend, clone C068C2, catalog no. 141715
	PE anti-mouse CD86	1/400, BioLegend, clone PO3, catalog no. 105105
	PE/Cyanine7 anti-mouse F4/80	1/200, BioLegend, clone BM8, catalog no. 123114
	PE/Dazzle 594 anti-mouse/human CD11b	1/ 800, BioLegend, clone M1/70, catalog no. 101255
	eBioscience Fixable Viability Due eFluor 506	1/800, Thermo Fisher Scientific, catalog no. 65-0866-18
T-cell markers	Brilliant Violet 570 anti-mouse CD4	1/400, BioLegend, clone RM4-5, catalog no. 100542
	Brilliant Violet 605 anti-mouse CD8a	1/400, BioLegend, clone 53-6.7, catalog no. 100744
	PE/Dazzle 594 anti-mouse CD279 (PD-1)	1/200, BioLegend, clone 29F.1A12, catalog no. 135228
	Alexa Fluor 488 anti-mouse FOXP3	1/100, BioLegend, clone MF-14, catalog no. 126406
	PerCP/Cyanine5.5 anti-mouse CD3ε	1/200, BioLegend, clone 145-2C11, catalog no. 100328
	Brilliant Violet 421 anti-mouse CD152 (CTLA4)	1/200, BioLegend, clone UC10-4B9, catalog no. 106312
	eBioscience Fixable Viability Due eFluor 506	1/800, Thermo Fisher Scientific, catalog no. 65-0866-18

### H&E Staining on PFA-fixed Cryosections

Ten-micron–thick sections were cut from unfixed tissues in a frozen block; sections were stored at −20°C until ready to use. When ready, slides were taken out of the freezer 30 minutes before staining and stained within an hour for optimal results. Room temperature slides were fixed with cold 4% PFA for 10 minutes at room temperature followed by washing in PBS for 10 minutes at room temperature. After washing, slides were dipped into Mayer's hematoxylin solution (see preparation under “Reagents and Solutions”) for 10 seconds, followed by washing in running tap water for 5 minutes. Then, slides were dipped into Eosin (5%) solution (see preparation under “Reagents and Solutions”) for 20 seconds, followed by washing in running tap water for 5 minutes. For rehydration, slides were placed first in 96% ethanol (2 × 2 minutes) followed by 100% ethanol (2 × 2 minutes). For clearance, slides were placed in RotiClear solution (Roth, catalog no. A538.5) for two times 5 minutes, after which time, slides were mounted using Eukitt quick-hardening mounting medium (Merck, catalog no. 03989). Slides were scanned using Leica DM6 B microscope and Leica Aperio Versa 8 slides scanner with 20× zoom and images were analyzed using the ImageScope program (version 12.3.3). QuPath was used to analyze the pulmonary tumor area coverage by dividing the tumor area per whole lung area and multiplying with 100. *N* = 6 lungs per group were analyzed.

### Statistical Analysis

All statistical analysis was carried out using one-way ANOVA and Fisher LSD (Least Significant Difference) tests, using the Statistica program (release 7), except for survival analysis, where GraphPad Prism (version 9.3.1) was used to perform Kaplan–Meier survival curves and Mantel–Cox for statistical analysis.

### Data Availability

All data needed to evaluate the conclusions on the article are presented in the article and/or the Supplementary Data. Additional data related to the findings of this study are available from the corresponding author.

## Results

### Design and Structural Modeling of St-PGA-OG-mUNO

**FIGURE 1:**
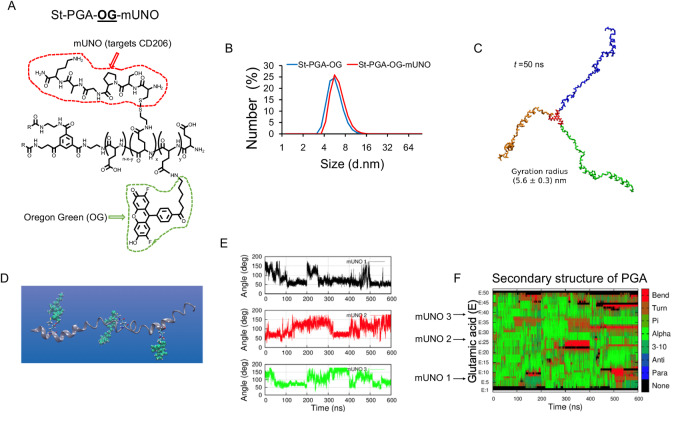
Design and analysis of mUNO-targeted St-PGA. **A,** Representative structure of St-PGA decorated with mUNO peptides (red) and OG (green). **B,** DLS graph demonstrating uniform size for both St-PGA-OG-mUNO and St-PGA-OG. **C,** A snapshot of modeled St-PGA structure in water and Na^+^ counterions at the last stage of the simulation (50 ns), displaying the three arms in different colors for visual clarity. The average gyration radius was 5.6 ± 0.3 nm, *t* shows time in ns. **D,** Representative MD snapshot of a single St-PGA-mUNO branch containing three equidistant mUNO peptides. Green spheres represent mUNO and a Licorice representation shows the linker. **E,** mUNO rotation around the PGA chain for each of the three peptides (black, red, and green lines). **F,** PGA chain secondary structure evolution, where red and brown regions show how mUNO perturbs the chain structure, turning alpha helices into random coils.

To characterize and explore the function of OximUNO, we first developed an mUNO-targeted St-PGA labeled with the OG fluorescent dye (referred to as St-PGA-OG-mUNO; Fig. 1A; Supplementary Scheme S1). We conjugated OG to St-PGA using an amide linker to allow *in vitro* or *in vivo* tracking and coupled mUNO through a disulphide bond formed between the free cysteine of mUNO and a pyridyldithiol linker on St-PGA. We previously demonstrated that mUNO conjugated to polymeric nanostructures through the cysteine thiol group preserves CD206 binding (42). To evaluate the structure and dye loading, we analyzed St-PGA-OG-mUNO and St-PGA-OG using NMR and UV-Vis analyses (Supplementary Fig. S1).

Dynamic light scattering (DLS) analysis demonstrated that St-PGA-OG-mUNO and St-PGA-OG displayed similar hydrodynamic diameters of 6.8 and 5.9 nm, respectively (Supplementary Table S1; Fig. 1B), while both nanoconjugates exhibited highly negative charges (−42 and −48 mV, respectively) as shown by Zeta potential analysis (Supplementary Table S1); an expected result given the glutamic acid nature of the polymer carrier. Analysis of mUNO loading (Supplementary Table S1) indicated the presence of approximately seven mUNO peptides in St-PGA-mUNO nanoconjugate, which would allow multivalent receptor binding.

We next assessed the structure of unlabeled and untargeted St-PGA in water using MD simulations to access information at the atomic scale. We assumed an initial helical conformation for the three PGA chains. The studied system consisted of a fully hydrated St-PGA and the Na^+^ counterions (∼920,000 atoms) and was built after initial minimization under vacuum conditions. We simulated 50 ns of the entire St-PGA macromolecule, with Fig. 1C displaying a snapshot corresponding to the last step of the simulation. Averaging the gyration radius over the last 25 ns of the simulation run provided a value of 5.6 ± 0.3 nm, which lies in the same order of magnitude as the results from DLS analysis and suggests a lack of aggregation of both St-PGA-OG-mUNO and St-PGA-OG in PBS. A video simulation (Supplementary Video S1) suggested that the three PGA chains remain in an extended conformation throughout the simulation and do not show any intramolecular or intermolecular interaction, suggesting that the mUNO peptides linked to St-PGA will not interfere with each other.

To investigate whether mUNO can engage with the CD206 receptor when grafted onto St-PGA, we modeled the structure and mobility of St-PGA-mUNO using computational analysis. To attain a computationally feasible system, we simulated only single branches of St-PGA-mUNO. We placed three equidistant mUNO peptides on a PGA single branch and fully solvated the system. We observed that three mUNO peptides remained exposed to the solution available for receptor binding (Fig. 1D). The rotation of mUNO around PGA, tracked by the angle formed by a proline aromatic carbon within mUNO (Supplementary Fig. S2, green sphere), a pyridyldithiol linker nitrogen (Supplementary Fig. S2, blue sphere), and a glutamic acid aromatic carbon (Supplementary Fig. S2, light blue sphere) revealed angles between 50° and 180° (Fig. 1E). This value supports the ability of mUNO peptides to interact with their receptor (43). Comparisons with an undecorated PGA branch demonstrated the minimal alterations of secondary structure dynamics in the presence of mUNO peptides—turning alpha helices (Fig. 1F, green) into random coils (Fig. 1F, brown) at regions where they are placed; however, the PGA chain structure remained mainly helical except in the middle, where a slight kink formed (Fig. 1F).

Altogether, St-PGA-OG-mUNO and St-PGA-OG nanoconjugates possessed similar sizes by DLS, highly negative charges, and, according to simulations, displayed their three arms in an extended open structure. Our simulation analyses demonstrated that mUNO peptides induced a minimal effect on PGA structure and rotated around the PGA chain with considerable freedom. Overall, these findings suggest St-PGA-mUNO as a suitable platform for CD206 targeting.

### St-PGA-OG-mUNO targets CD206^+^ TAMs and Displays Low Hepatic Accumulation

We next evaluated the potential of St-PGA-OG-mUNO to target CD206^+^ TAMs in a TNBC model—induced by orthotopic inoculation (referred to as “orthotopic TNBC”) or by intravenous inoculation (referred to as “experimental metastasis of TNBC”) of 4T1 cells. We administered St-PGA-OG-mUNO or St-PGA-OG intraperitoneally, allowed circulation for 6 hours, and then analyzed tumor homing using confocal fluorescence microscopy. Our previous study provided the rationale for the intraperitoneal administration route, where we demonstrated that the intraperitoneally administered mUNO peptide exhibited a substantially longer half-life than intravenously administered mUNO in the same mice (same strain, sex, and age) used in this study (42).

**FIGURE 2:**
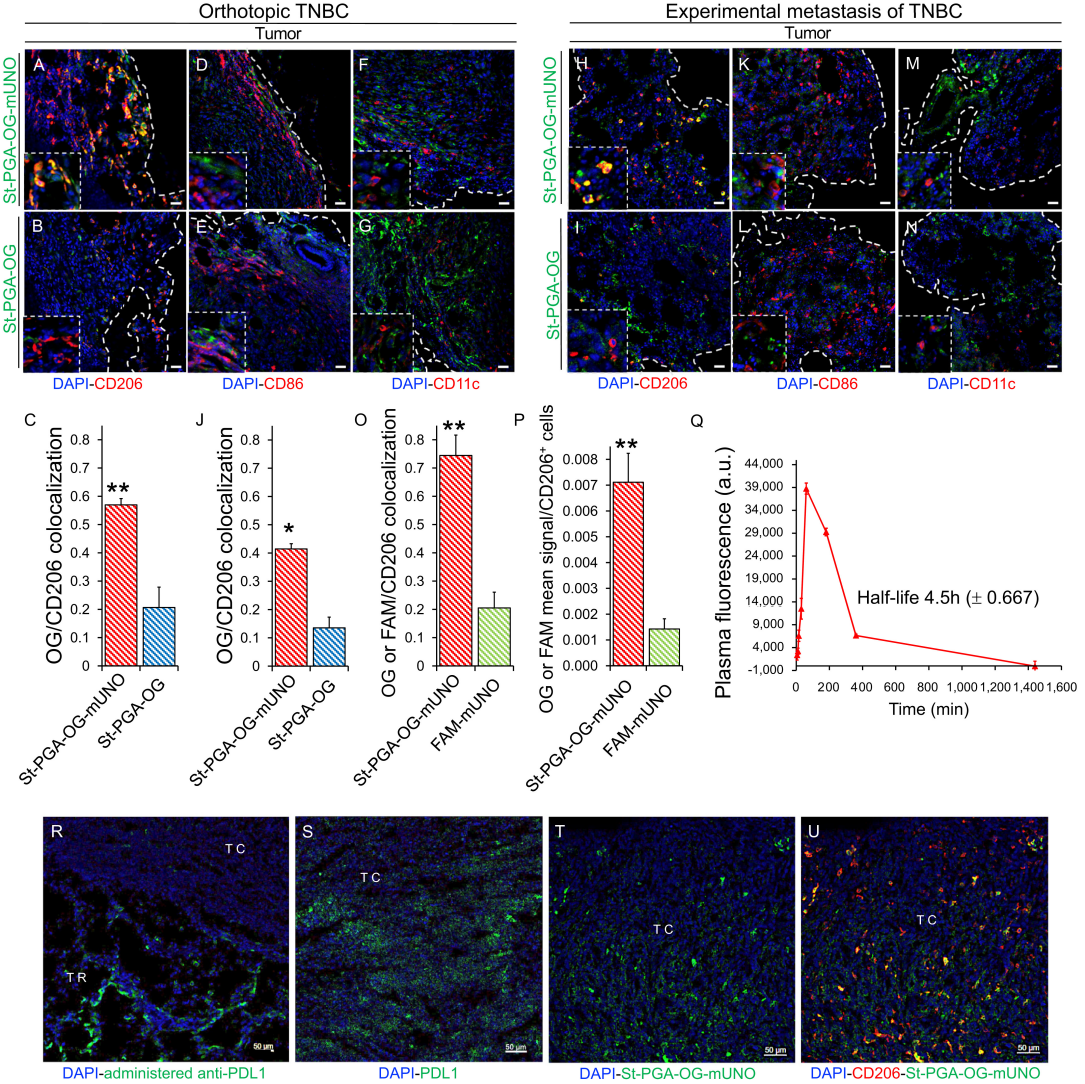
St-PGA-OG-mUNO targets CD206^+^ TAMs in models of orthotopic TNBC and experimental metastasis of TNBC and displays an extended plasma half-life. Homing studies with intraperitonally administered St-PGA-OG-mUNO (0.41 mg/0.5 mL of PBS) or St-PGA-OG (0.35 mg/0.5 mL of PBS), after 6 hours of circulation. *N* = 3 for orthotopic TNBC and *N* = 2 for experimental metastasis of TNBC. **A–G,** Homing in orthotopic TNBC. **A,** St-PGA-OG-mUNO displayed high colocalization between OG and CD206 (yellow signal), whereas St-PGA-OG displayed minimal colocalization (**B**). **C,** Graph depicting the quantification of CD206 and OG colocalization in the orthotopic TNBC. St-PGA-OG-mUNO and St-PGA-OG did not show any homing to CD86^+^ cells (M1 macrophages; **D** and **E**) nor CD11c^+^ cells (DCs; **F** and **G**). **H–N**, Homing study in the experimental metastasis of TNBC. St-PGA-OG-mUNO displayed high colocalization with OG and CD206 (yellow signal; **H**), whereas St-PGA-OG showed minimal colocalization (**I**). **J,** Graph depicting the quantification of CD206 and OG colocalization in the experimental metastasis of TNBC. St-PGA-OG-mUNO and St-PGA-OG did not show any homing to CD86^+^ cells (M1 macrophages; **K** and **L**) or CD11c^+^ cells (DCs; **M** and **N**). Scale bars = 20 μm. **O,** Quantification of colocalization analysis for St-PGA-OG-mUNO or FAM-mUNO with CD206 homing after 6 hours of circulation, *N* = 2 (30 nanomoles in OG and FAM, respectively). Colocalization was quantified using the Fiji program and Pearson coefficient (for more information, see Materials and Methods). **P,** Mean OG/FAM signal per CD206^+^ cell analyzed using the ImageJ program. **Q,** Plasma fluorescence (in the green channel) of intraperitoneallly administered St-PGA-OG-mUNO (dose 15 nanomoles in OG) in healthy Balb/c mice (*N* = 3). **R,** Rat anti-mouse PD-L1 was intravenously injected 10 days after tumor induction (p.i.) and circulated for 24 hours after which time, mice were sacrificed, tumors collected, fixed, and stained for rat IgG. **S,** PDL1 expression was detected in noninjected subcutaneous 4T1 tumors by staining with rat anti-mouse PDL1. **T** and **U,** Representative images showing St-PGA-OG-mUNO (0.41 mg/0.5 mL) intraperitoneally injected 10 days after tumor induction and circulated for 6 hours after which time, mice were sacrificed, tumors fixed, and then stained for OG and CD206. The OG channel is shown separately in **T**, and the colocalization with CD206 for the same image is shown in **U**. Scale bars = 50 μm. Error bars represent SEM. *, *P* ≤ 0.05; **, *P* ≤ 0.01.

In the orthotopic TNBC, we observed a high colocalization of OG/CD206 (Fig. 2A, yellow signal) with St-PGA-OG-mUNO but a much lower colocalization of OG/CD206 with nontargeted St-PGA-OG (Fig. 2B) [0.57 and 0.21, respectively (Fig. 2C)]. We observed a low level of accumulation of St-PGA-OG-mUNO or St-PGA-OG in the liver (Supplementary Fig. S3A and S3B). We employed confocal image acquisition parameters throughout this study to visualize CD206 in the tumor without signal saturation. Given the higher levels of CD206 in the tumor, imaging with associated settings provides low CD206 visualization in the liver. Using a higher image intensity, we observed the expected CD206 signal in the liver (as expected from Kupffer cells and sinusoid vessels; Supplementary Fig. S4A) and a saturated CD206 signal in the tumor (Supplementary Fig. S4B).

Immunostaining for endogenous mouse IgG in the tumor and the liver indicated the leaky nature of the tumor vasculature (Supplementary Fig. S5A) compared with the liver vasculature (Supplementary Fig. S5B) in the 4T1 model. A leaky tumor vasculature favors the hypothesis that St-PGA-OG-mUNO has a more extended (both in time and space) access to CD206 in the tumor than in the liver. We speculate that the leaky tumor vasculature combined with lower CD206 expression in the liver than the tumor explains the low hepatic accumulation of St-PGA-OG-mUNO. St-PGA-OG-mUNO did not accumulate in the lungs (Supplementary Fig. S6A) or spleen (Supplementary Fig. S6B); however, we did observe some accumulation in the sentinel lymph node (Supplementary Fig. S6C) and the kidneys (Supplementary Fig. S6D). Of note, the observed kidney signal agrees with our prior studies that demonstrated the renal excretion of St-PGA (53).

Importantly, we did not detect homing to M1 macrophages (CD86^+^) or dendritic cells (CD11c^+^, DC) with St-PGA-OG-mUNO or with St-PGA-OG (Fig. 2D–G). In the experimental metastasis of TNBC, most of the cellular signal of St-PGA-OG-mUNO associated with CD206^+^ TAMs (Fig. 2H, yellow signal) when compared with St-PGA-OG (Fig. 2I; OG/CD206 colocalization 0.42 and 0.14, respectively, Fig. 2J). Here, we also observed no colocalization between OG and CD86 (M1 macrophages; Fig. 2K and L) or OG and CD11c (DCs; Fig. 2M and N) and the observed hepatic accumulation of St-PGA-OG-mUNO or St-PGA-OG was low (Supplementary Fig. S7).

One of the rationales behind the design of OximUNO was to increase mUNO targeting through increased avidity and plasma half-life. To evaluate these aspects, we compared the homing of St-PGA-OG-mUNO with a monomeric, carboxyfluorescein-labeled mUNO peptide (FAM-mUNO). We note that even given the different nature of the fluorescent labels (OG on St-PGA-OG-mUNO and fluorescein on FAM-mUNO), we did not use their native fluorescence as a readout; instead, we used an antibody that recognizes both FAM and OG; therefore, we do not expect biases from potential differences in FAM and OG emissions.

We discovered that St-PGA-OG-mUNO (Supplementary Fig. S8A) displayed significantly higher OG/CD206 colocalization than for FAM/CD206 with FAM-mUNO (Supplementary Fig. S8B) at 6 hours [0.74 vs. 0.21, respectively (Fig. 2O)]. In addition, we found that the OG/FAM mean signal per CD206^+^ cell was five times higher for St-PGA-OG-mUNO than FAM-mUNO (Fig. 2P). These findings suggest that conjugating mUNO to the St-PGA backbone greatly improved receptor binding.

Plasma half-life analysis for intraperitoneally administered St-PGA-OG-mUNO revealed a 4.5-hour half-life (Fig. 2Q), a value over two times longer than that observed after the intraperitoneal administration of FAM-mUNO in our previous study (42). We previously showed that the plasma half-life of systemically administered St-PGA is approximately 12 hours (53), that negligible degradation of FAM coupled to mUNO through an amide bond (FAM-mUNO) occurs in serum (42), and that the fluorescence of FAM-UNO was not affected by serum from mice bearing 4T1 tumors (41). On the basis of these antecedents, we here attributed the plasma fluorescence of Fig 2Q, to St-PGA-OG-mUNO.

Overall, this finding suggests that conjugating mUNO to St-PGA increased the plasma half-life of mUNO peptide, a desirable feature that will improve *in vivo* ligand targeting.

We next compared tumor homing of St-PGA-OG-mUNO with that of a therapeutic mAb by intravenously injecting anti-PDL1 in orthotopic 4T1 tumor-bearing mice and allowing circulation for 24 hours. We observed that administered anti-PDL1 accumulated in the tumor rim (Fig. 2R, TR) but not in the tumor core (Fig. 2R, TC) even given expression of the receptor (PDL1) in the tumor core (Fig. 2S, TC). The observed accumulation of St-PGA-OG-mUNO in the tumor core (Fig. 2T, TC) and receptor colocalization (Fig. 2U), supported the implementation of our platform as an efficient alternative to antibody-based therapies such as anti-PDL1 or antibody–drug conjugates.

Administration of a higher dose of nanoconjugate (0.82 mg/0.5 mL St-PGA-OG-mUNO and 0.7 mg/0.5 mL St-PGA-OG) resulted in high CD206^+^ TAM targeting for St-PGA-OG-mUNO (Supplementary Fig. S9A) albeit at the expense of higher hepatic accumulation (Supplementary Fig. S9B). Tumor and hepatic accumulation of St-PGA-OG are shown in Supplementary Fig. S9C and S9D. For this reason, we employed lower nanoconjugate doses (0.41 mg/0.5 mL and 0.35 mg/0.5 mL) for subsequent studies. As St-PGA-OG-mUNO did not target the lung, liver, or spleen (Supplementary Fig. S6), at this dose we expect OximUNO not to affect the macrophage populations of those organs.

Overall, we demonstrated that St-PGA-OG-mUNO homes to CD206^+^ TAMs in the orthotopic TNBC and in experimental metastasis of TNBC, with no significant hepatic accumulation. We also established that St-PGA-OG-mUNO does not target M1 macrophages or DCs in the tumor, thereby providing evidence of high specificity for CD206^+^ TAMs.

### OximUNO Enhances the *In Vitro* Cytotoxicity of DOX on M2-resembling Macrophages

**FIGURE 3:**
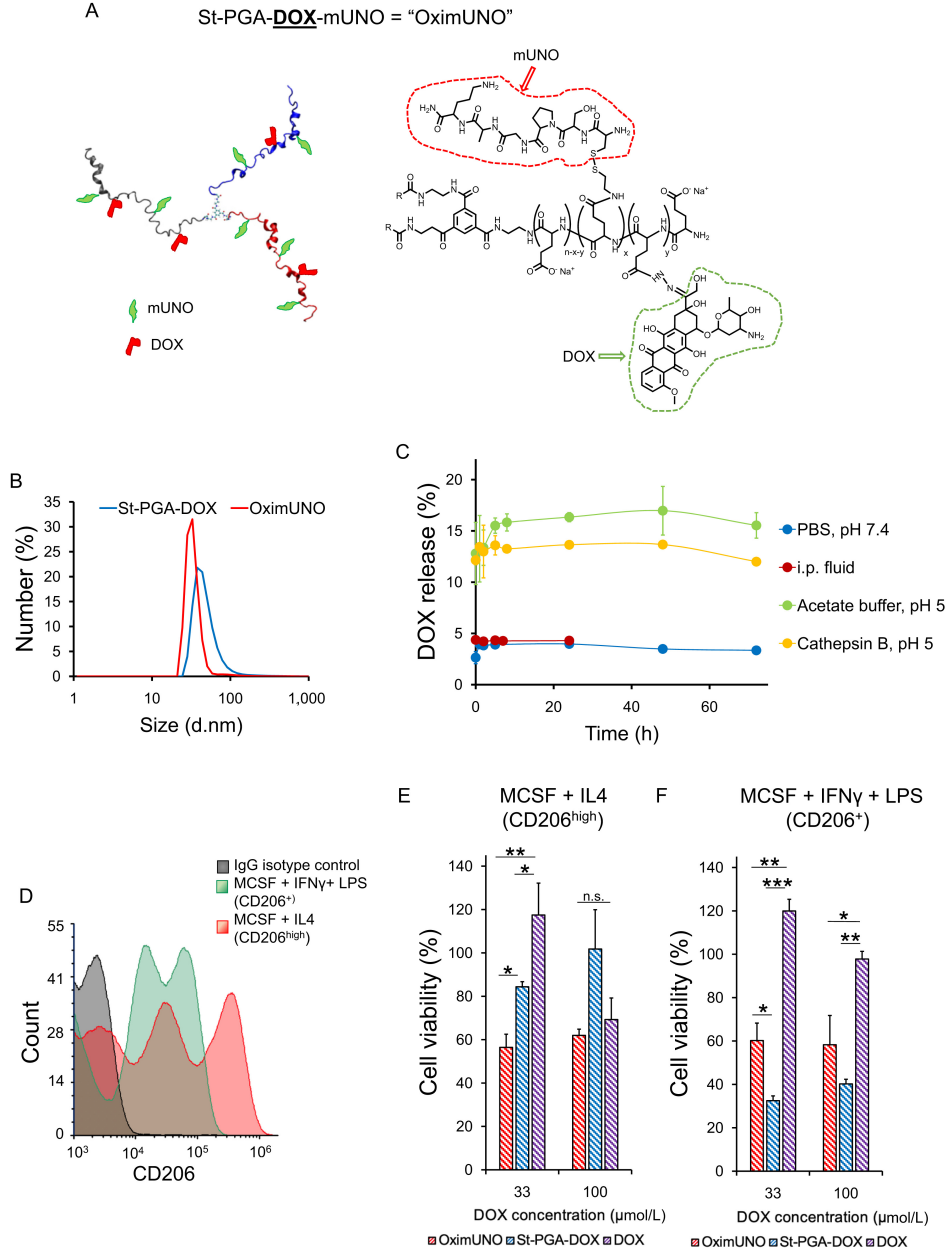
OximUNO enhances the *in vitro* efficacy of DOX on M2-resembling macrophages. **A,** Simplified form of OximUNO (left) and molecular structure (right) showing St-PGA decorated with mUNO (red) and DOX (green). **B,** A DLS graph for measurements shown in Supplementary Table S2, indicating the uniform size of OximUNO and St-PGA-DOX. **C,** DOX release from OximUNO showing the drug release in PBS, intraperitoneal fluid, acetate buffer or in the presence of cathepsin B. **D,** CD206 expression by M1-resembling (green) and M2-resembling (red) macrophages using FC. *In vitro* cytotoxicity in primary human M2-resembling (MCSF + IL4 polarized; **E**), M1-resembling (MCSF + IFNγ + LPS polarized; **F**) macrophages after treatment with OximUNO (red bars), St-PGA-DOX (blue bars), and DOX (purple bars) following a 15-minute incubation, washed, cultured for additional 48 hours, and then analyzed for cell viability as evaluated by MTT assay. Error bars represent SEM. *, *P* ≤ 0.05; **, *P* ≤ 0.01; ***, *P* ≤ 0.001.

St-PGA displays a large surface with multiple sites available for the conjugation of proapoptotic or cytotoxic cargoes via bioresponsive polymer-drug linkers (54, 55). To selectively deplete CD206^+^ TAMs, we conjugated an apoptotic chemotherapeutic agent (DOX) to St-PGA-mUNO to form St-PGA-DOX-mUNO (designated “OximUNO”; Fig. 3A, Scheme S2). We conjugated DOX to St-PGA-mUNO using a hydrazone bond (54) to allow for site-specific drug release in the acidic milieu of the endosomes or lysosomes (54, 56).

To evaluate the effect of mUNO targeting, we included St-PGA-DOX as an untargeted control. We employed ^1^H NMR and UV-Vis analyses to evaluate the chemical identity of nanoconjugates (Supplementary Fig. S10A and S10B).

OximUNO displayed DOX and mUNO loadings of approximately 10% and approximately 4% in weight, respectively, corresponding to around four DOX and seven mUNO molecules for every OximUNO. OximUNO exhibited a size of approximately 40 nm and a highly negative surface charge of −40 mV (Supplementary Table S2; Fig. 3B). We obtained similar DOX loading, size by DLS, and surface charge values for St-PGA-DOX (Supplementary Table S2; Fig. 3B).

The pH-sensitive hydrazone linker and the intrinsic biodegradability of St-PGA by lysosomal protease cathepsin B are expected to secure DOX release from OximUNO after cell internalization (57). Hence, we studied DOX release kinetics from OximUNO in the presence of acidic pH (pH 5) and cathepsin B using LC/MS (Supplementary Fig. S11A–S11G). As we aimed for the intraperitoneal administration of OximUNO, we assessed DOX release in intraperitoneal fluid (Fig. 3C). At pH 5, we observed a sustained DOX release in the first 8 hours (reaching a plateau at 15%), thereby demonstrating the suitability for endosomal-lysosomal drug delivery. DOX release in the presence of cathepsin B displayed comparable values in the first 8 hours (∼13%), followed by a plateau and a reduced rate in the following hours (∼13% cumulative release at 72 hours). Importantly, OximUNO exhibited negligible drug release in both physiologic conditions evaluated (PBS and intraperitoneal fluid; Fig. 3C).

We next evaluated the *in vitro* cytotoxicity of OximUNO and St-PGA-DOX in primary human macrophages derived from PBMCs, polarized with different cytokines to resemble M2 (MCSF + IL4) and M1 (MCSF + IFNγ + LPS) macrophages. Under these conditions, macrophages polarized with MCSF + IFNγ + LPS expressed nonnegligible CD206 levels, albeit at levels lower than for macrophages polarized with MCSF + IL4 (Fig. 3D). Bertani and colleagues (58) observed the same pattern of CD206 expression in PBMC-derived macrophages polarized under similar conditions.

Because the *in vivo* concentration that provided optimal CD206^+^ TAM targeting with minimal hepatic accumulation was 30 μmol/L in OG, here we focused our interest on conjugates at 33 μmol/L of DOX. Our previous studies comparing other mUNO-targeted versus untargeted polymeric nanosystems (44) demonstrated that the highest targeted uptake in primary MCSF + IL4 polarized macrophages occurred after an interval of 10 to 30 minutes. For this reason, we used an incubation time of 15 minutes for these experiments.

Importantly, in MCSF + IL4 polarized macrophages, OximUNO displayed a significantly higher toxicity than DOX and St-PGA-DOX (Fig. 3E). St-PGA-DOX showed its highest toxicity in MCSF + IFNγ + LPS polarized macrophages (Fig. 3F). We speculate that here, the phagocytic activity, known to be highest for MCSF + IFNγ + LPS polarized macrophages (59, 60), governs the uptake of St-PGA-DOX. Here, the 60% cell viability observed for OximUNO (Fig. 3F) is consistent with the fact that under these conditions, MCSF + IFNγ + LPS polarized macrophages also expressed CD206 (Fig. 3D). *In vivo*, St-PGA-OG-mUNO did not target M1 TAMs (Fig. 2D and K); hence, we do not expect OximUNO to affect this population. Free DOX only displayed toxicity in MCSF + IL4 polarized macrophages at 100 μmol/L (Fig. 3E).

These results provide evidence that OximUNO displayed increased toxicity toward M2-resembling macrophages when compared with St-PGA-DOX or DOX alone.

We also evaluated the hepatic and renal safety profile of a single administration of OximUNO (at doses corresponding to 2 and 4 mg/kg of DOX) by analyzing creatinine (Crea) and alanine aminotransferase (ALAT) levels 48 hours after intraperitoneal administration in healthy mice (Supplementary Table S3). These doses did not induce toxic levels of Crea or ALAT compared with the values reported in the literature (61) or the reference values for the female Balb/c reported in the Mouse Phenome Database by The Jackson Laboratory (https://phenome.jax.org/search/details/ssmeasures?searchterm=alanine+aminotransferase+&ontavail=2) or Charles River facilities (https://www.criver.com/products-services/find-model/balbc-mouse?region=3616). However, increased ALAT levels with the higher dose prompted the selection of the OximUNO dose corresponding to 2 mg/kg of DOX for further *in vivo* studies. Administration of OximUNO at 2 mg/kg of DOX showed no histologic changes indicative of hepatic and renal toxicity, as evaluated by a pathologist (Supplementary Fig. S12A), and IFNγ IF did not detect a clear increase that would indicate inflammatory changes (Supplementary Fig. S12B).

In summary, the conjugation of mUNO and DOX to the St-PGA backbone to yield OximUNO, enhanced the *in vitro* efficacy of DOX toward M2-resembling macrophages with no *in vivo* renal or hepatic toxicity observed.

### OximUNO Treatment of Orthotopic TNBC Depletes CD206^+^ TAMs, Inhibits Tumor Progression, and Attenuates Immunosuppression

**FIGURE 4:**
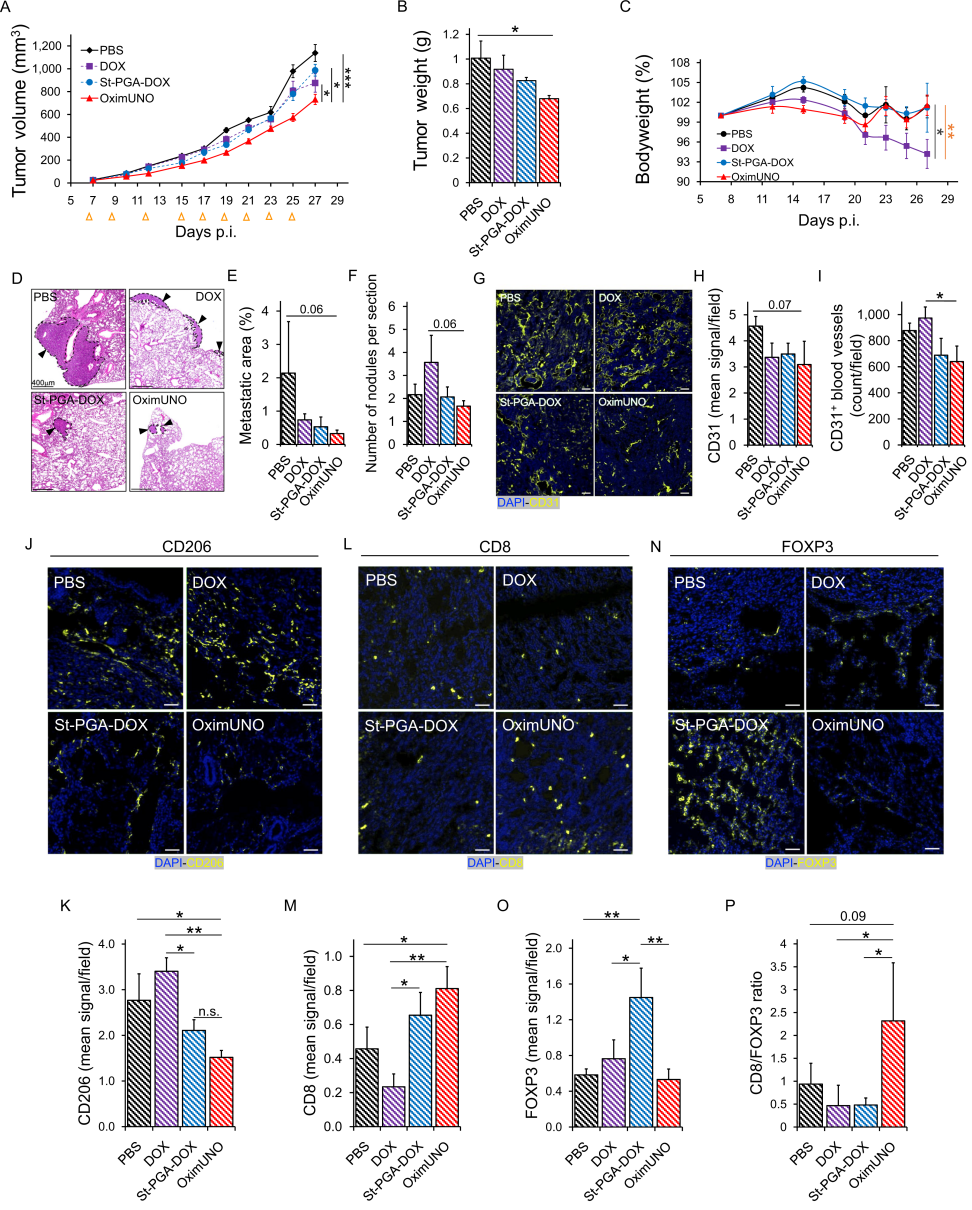
OximUNO treatment reduces primary tumor growth and pulmonary metastases and alleviates immunosuppression. Treatment with OximUNO, St-PGA-DOX, or DOX at 2 mg/kg of DOX in mice bearing orthotopic TNBC tumors (*N* = 5). Intraperitoneal injections began when tumors reached 25 mm^3^ and were performed every other day to give a total of nine injections. **A,** Primary tumor volume progression during treatment. Orange arrows indicate injection days. **B,** Primary tumor weight at the experimental endpoint, demonstrating a significantly smaller weight for OximUNO-treated mice (red bar) than other groups. **C,** Mouse bodyweight analysis suggests the safety of OximUNO treatment (red line); meanwhile, DOX treatment induced a significant reduction in bodyweight by the experimental endpoint (purple line). Dark gray ∗ DOX versus PBS, orange ∗ DOX versus St-PGA-DOX. **D,** Representative H&E images showing spontaneous pulmonary metastases for all groups (scale bars = 400 μm); OximUNO treatment associated with the smallest metastatic area (**E**) and the lowest number of average nodules per lung (**F**). **G,** Representative images showing the expression of CD31 and blood vessels in primary tumors. **H,** Graph depicting the expression of CD31 in primary tumors. **I,** Graph depicting the CD31^+^ blood vessel count in primary tumors. CD31 expression and blood vessel count were calculated using ImageJ and five images per mouse per group for expression analysis and at least three images per mouse per group for blood vessel count. Representative confocal microscopy images and quantification graphs of primary tumors demonstrating the expression of CD206 (**J** and **K)**, CD8 (**L** and **M**), and FOXP3 (**N** and **O**). Scale bars = 50 μm. **P,** Graph of CD8/FOXP3 expression ratio showing a shift in the immune profile. Quantification was performed using the ImageJ from at least three images per mouse and 5 mice per group. Error bars represent SEM. *, *P* ≤ 0.05; **, *P* ≤ 0.01; ***, *P* ≤ 0.001; n.s., > 0.05.

The findings of the *in vivo* homing and *in vitro* cytotoxicity studies supported the subsequent evaluation of OximUNO in the orthotopic TNBC. When tumors reached 25 mm^3^, we treated mice with intraperitoneal injections of OximUNO, St-PGA-DOX, or DOX, at 2 mg/kg of DOX every other day for 18 days. Encouragingly, OximUNO treatment significantly reduced primary tumor volume growth kinetics (Fig. 4A, red line) compared with DOX, St-PGA-DOX, and PBS. Furthermore, only the OximUNO treatment significantly reduced final tumor weight (Fig. 4B) compared with the untreated group. We assigned this encouraging therapeutic effect to mUNO-mediated targeting, as mice treated with the untargeted St-PGA-DOX possessed tumor volumes (Fig. 4A, blue line) similar to the PBS group (Fig. 4A, black line). Furthermore, OximUNO treatment did not affect mouse bodyweight, whereas treatment with DOX induced a significant decrease in mouse bodyweight starting from day 21 p.i. until the end of the treatment (Fig. 4C).

Histologic analysis of lungs from treated mice (Supplementary Fig. S13 shows an H&E stain from a healthy lung for comparison) revealed that OximUNO showed a decreasing trend in the metastatic lung area and nodule number (Fig. 4D–F). Meanwhile, IF microscopy revealed no significant changes in CD31 expression in tumors (Fig. 4G and H), but significantly fewer CD31^+^ structures in the OximUNO-treated mice compared with DOX-treated mice (Fig. 4G and I), suggesting that the reduction in nodule number in the OximUNO group (of Fig. 4F) may be mediated by the lower vascularization in the primary tumor. We suggest that partial vascular homing of St-PGA-DOX in the tumor (as suggested by the tumor homing of its OG equivalent; Supplementary Fig. S14) contributes to the blood vessel reduction observed in this group. Importantly, histologic analysis revealed no cardiotoxicity in any treatment groups (Supplementary Fig. S15). IF analysis revealed that only OximUNO significantly reduced the CD206 expression (assigned to CD206^+^ TAMs), compared with PBS (Fig. 4J and K). Interestingly, treatment with DOX upregulated CD206 expression (Fig. 4J and K), which agrees with previous reports that demonstrated an increase in the number of CD206^+^ TAMs following chemotherapy (24).

Notably, only OximUNO treatment significantly increased CD8 expression [a marker of cytotoxic T lymphocyte (CTL)] compared with PBS and DOX treatment (Fig. 4L and M). Unexpectedly, St-PGA-DOX treatment increased the expression of FOXP3, a marker for regulatory T cells (Treg; Fig. 4N and O). Analysis of the CD8/FOXP3 expression ratio revealed that OximUNO treatment resulted in a 5-fold increase compared with St-PGA-DOX or DOX treatment (Fig. 4P), suggesting that OximUNO stimulated a shift in the immune landscape toward immunostimulation. Of note, in all cases, we normalized the quantification of marker expression using immunofluorescent images to the tissue area to account for different amounts of tissue in different images.

A repetition of this treatment study, monitoring primary tumor growth and survival, showed the slowest tumor growth kinetics in the OximUNO group (Supplementary Fig. S16A) and Kaplan–Meier curves showed a significantly prolonged survival for OximUNO-treated mice compared with untreated mice (Supplementary Fig. S16B–S16D).

By targeting CD206^+^ TAMs with DOX via OximUNO treatment, we increased the efficacy and reduced the toxicity of DOX in the orthotopic TNBC. Our results also suggest that the depletion of CD206^+^ TAMs by OximUNO elicited an immunostimulatory shift.

### OximUNO Treatment of Experimental Metastasis of TNBC Reduces CD206^+^ TAMs Number, Tumor Burden and Attenuates Immunosuppression

**FIGURE 5:**
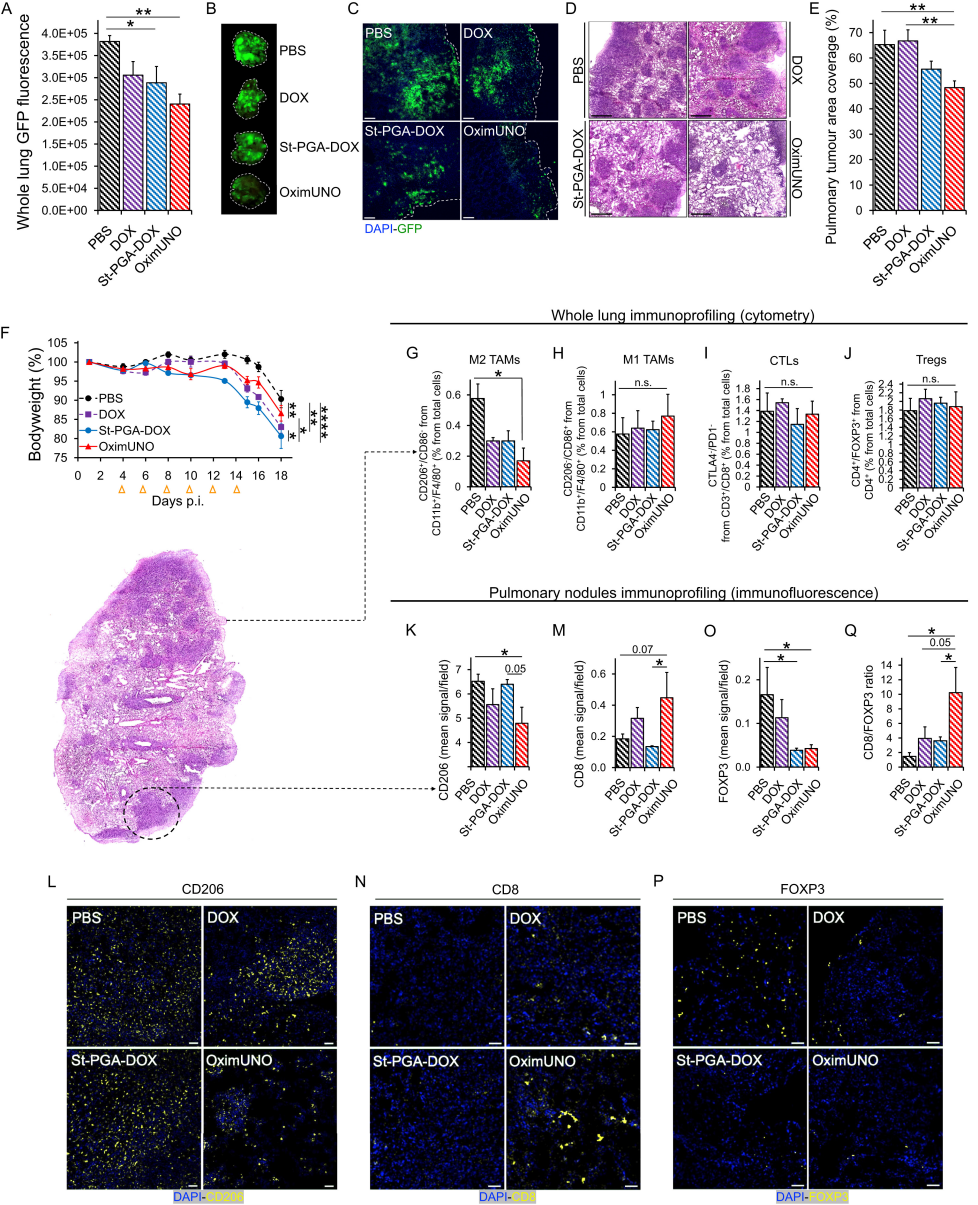
OximUNO treatment in experimental metastasis of TNBC significantly reduces CD206^+^ TAM number and tumor burden and alleviates immunosuppression. Treatment with OximUNO, St-PGA-DOX, or DOX at 2 mg/kg of DOX in the experimental metastasis of TNBC model, created using GFP-labeled 4T1 cells (*N* = 6). Intraperitoneal injections began on day 4 p.i. and were performed every other day to give a total of six injections. **A,** Quantification of whole lung GFP fluorescence at the experimental endpoint using the ImageJ (*N* = 6). **B,** Representative macroscopic photographs of GFP fluorescence in the lungs. **C,** Representative confocal microscopy images of GFP expression, scale bars = 100 μm. **D,** Representative H&E images showing pulmonary metastases for all groups (scale bars = 900 μm). **E,** Quantification of pulmonary metastases from H&E images, expressed as percentual area of whole lung section. **F,** Mouse bodyweight analysis, demonstrating significantly lower bodyweight lost with OximUNO (red line) compared with St-PGA-DOX–treated mice (blue line) and DOX-treated mice (purple dotted line). Orange arrows indicate injection days. **G–J**, FC analysis on three right lungs per group. M2 TAMs (CD206^+^; **G**), M1 TAMs (**H**), CTLs (**I**) and Tregs (**J**). Representative IF images and analysis on the pulmonary tumor nodules to detect the expression of CD206 (**K** and **L**), CD8 (**M** and **N**), and FOXP3 (**O** and **P**). **Q,** Graph showing CD8/FOXP3 expression ratio. IF images quantified using the ImageJ from at least five images per mouse and 3 mice per group. Scale bars = 50 μm. Error bars represent SEM. *, *P* ≤ 0.05; **, *P* ≤ 0.01; ****, *P* ≤ 0.0001; n.s., > 0.05.

We next evaluated the effect of OximUNO on experimental metastasis of TNBC using GFP-labeled 4T1 cells. We treated mice every other day with intraperitoneal injections of OximUNO, St-PGA-DOX, or DOX, starting from day 4 p.i. and sacrificed mice on day 18 p.i. Analysis of whole lung fluorescence in the green channel revealed that OximUNO treatment induced the lowest GFP fluorescence, indicating a lower level of pulmonary metastases (Fig. 5A). Representative macroscopic images also provided evidence for a reduction in metastases (Fig. 5B). Confocal fluorescence microscopy of lungs confirmed the trend observed with whole lung fluorescence, showing fewer GFP fluorescent nodules in the OximUNO-treated group (Fig. 5C). Furthermore, histologic analysis of lungs displayed the lowest number of pulmonary nodules for OximUNO-treated mice (Fig. 5D and E). Mice treated with the untargeted St-PGA-DOX and free DOX showed a significant decrease in bodyweight, resulting in a 19% (Fig. 5F, blue line) and 17% loss (Fig. 5F, purple line), respectively; meanwhile, OximUNO-treated mice displayed lower bodyweight loss (Fig. 5F, red line).

We next employed FC to analyze the effect of different treatments on the immune cell populations in whole lungs. This analysis demonstrated that OximUNO treatment significantly lowered the percentage of M2 TAMs (CD206^+^; Fig. 5G) but did not significantly impact the percentage of M1 TAMs, CTLs, or Tregs (Fig. 5H–J). We observed the same trend when we expressed these populations as total cell counts (Supplementary Fig. S17–S20).

To evaluate whether OximUNO affected CD206^+^ macrophages other than M2 TAMs, we analyzed the state of splenic macrophages from this treatment study using FC. This analysis revealed no significant differences in the CD206/CD86 populations between the OximUNO-treated mice and PBS-treated mice (Supplementary Fig. S21A–S21C).

While FC analysis informs on the immune status of the whole lung, it does not provide specific information regarding the TME. To characterize the immune landscape of the TME, we next analyzed the expression of markers for TAMs, CTLs, and Tregs in pulmonary nodules using IF. This analysis revealed significantly lower CD206 expression in OximUNO-treated mice than PBS-treated mice (Fig. 5K and L), providing evidence for a robust reduction in the number of CD206^+^ TAMs in the TME. Importantly, and similarly to OximUNO treatment in the orthotopic TNBC, OximUNO elicited the highest expression of CD8 (Fig. 5M and N). OximUNO-treated and St-PGA-DOX–treated mice demonstrated significantly lower lung FOXP3 expression than PBS- and DOX-treated mice (Fig. 5O and P). OximUNO-treated mice displayed between a two and three times higher CD8/FOXP3 expression ratio than St-PGA-DOX and DOX, and nearly seven times higher than PBS (Fig. 5Q). Therefore, our IF analysis in the pulmonary tumor nodules suggested that OximUNO triggered a shift in the immune profile of the TME toward immunostimulation.

By targeting DOX to CD206^+^ TAMs in experimental metastasis of TNBC, we increased the efficacy and reduced the toxicity of DOX, as OximUNO treatment associated with the presence of fewer pulmonary tumor lesions and less bodyweight loss when compared with treatment with untargeted St-PGA-DOX and DOX. Our results suggest that the observed therapeutic effect derived from CD206^+^ TAM depletion, which elicited an immunologic shift in the TME.

## Discussion

To date, TNBC remains an aggressive breast cancer subtype (3) with few treatment options, with conventional chemotherapy representing the current standard of care (20). ICIs for TNBC have provided only modest improvements in complete response and progression-free survival in a small subset of patients with TNBC (9, 12, 15, 16). Targeting TAMs can potentiate ICIs and other modalities and, therefore, represents an intense area of study (62–66); however, TAMs represent a diverse population (67–69), and which TAM subtype to target remains under investigation.

Promising TAM-focused interventions under clinical evaluation include antibody-mediated depletion of TREM2-expressing TAMs (clinical trial identifier: NCT04691375). Antibody blockade of Clever-1 on M2 TAMs stimulated an M2→M1 switch in TNBC models (4T1) and synergized with the PD-1 blockade (70). Appealing studies have used anti-CD163 antibodies to target TAMs (71) by decorating DOX-carrying liposomes with anti-CD163, to deplete TAMs and potentiate ICIs in melanoma. Given our data comparing the tumor penetration of an anti-PDL1 antibody versus St-PGA-OG-mUNO, anti-CD163 systems may also display lower tumor accumulation than St-PGA-OG-mUNO and OximUNO. Strategies targeting generic TAM markers such as CSF1R and CCR2 have shown side effects and limited efficacy.

Motivated by the preponderance of the mannose receptor in tumorigenic/metastatic TAMs in breast cancer (72–74), here, we set out to deplete CD206^+^ TAMs in an aggressive TNBC model and study the consequences on the progression and immunosuppressive state of the tumor. To target CD206, a CD206-binding nanobody was developed by Ginderachter and colleagues (75) which showed homing to CD206^+^ TAMs in *in vivo* models of lung and breast cancers (75). Navidea Inc. engineered a mannosylated compound (ref. 76; Manocept), that forms part of the FDA-approved contrast agent Lymphoseek. Unfortunately, mannose-based ligands have other binding partners besides CD206, including CD209 in intestinal and genital tissues (45), and can target dendritic cells (46). Riptide Inc. also designed a peptide (RP-182) that binds to CD206; however, the peptide also binds to RelB, Sirp-α, and CD47 (77).

We recently identified and described a short peptide called mUNO (sequence: CSPGAK) that targets mouse (41) and human CD206 (43) at a different binding site than for mannose on CD206 (43). We identified mUNO from an *in vivo* screen using a peptide library in mice bearing metastatic breast cancer; we subsequently described how mUNO homed to CD206^+^ TAMs in other solid tumor models (41, 78) and in early-stage models of TNBC (42) displaying low hepatic accumulation.

We envisioned that conjugating mUNO to St-PGA would significantly enhance targeting through the avidity effect and increased plasma half-life (79).

Compared with synthetic polymers such as N-(2-hydroxypropyl) methacrylamide, polypeptide-based nanocarriers show several benefits, including biodegradability, lower immunogenicity, and a lack of long-term accumulation, and the number of polypeptide-based constructs reaching clinical evaluation has significantly increased in recent years (80–82). We employed St-PGA–based nanoconjugates with three linear chains (∼50 glutamic acids each) linked to a central core. Overall, the safety, lack of toxicity, and biodegradability of St-PGA meet FDA approval criteria (83). A previous screen of PGA structures suggested that larger architectures enhanced plasma half-life and increased bioavailability through a higher hydrodynamic volume that reduces rapid renal clearance (53, 84). Of note, an extended plasma half-life will be advantageous when targeting the continuously replenished TAM cell type (85, 86).

St-PGA-OG-mUNO, a fluorescent counterpart of OximUNO, can be easily monitored by immunostaining for OG or detecting native OG fluorescence (as for the half-life study). Given weak DOX fluorescence and the inability to detect DOX with an antibody, we first designed St-PGA-OG-mUNO for validation purposes. We then exchanged OG for DOX to generate St-PGA-DOX-mUNO, referred to as “OximUNO.” Our studies demonstrated that St-PGA-OG-mUNO displayed a far greater plasma half-life and specificity to CD206^+^ TAMs than free mUNO and avoided CD86^+^ M1 TAMs and CD11c^+^ DCs, an important fact because M1 TAMs display antitumorigenic activity (25), and CD11c^+^ DCs participate in antigen presentation (87). In line with these observations, the computational analysis indicated that mUNO peptides are available to a receptor and sweep a vast space (130°) around PGA. Altogether these data demonstrate the benefit of conjugating mUNO to St-PGA. While previous studies have reported the St-PGA nanocarrier (53, 83) and the mUNO-targeting peptide (42), this work represents a novel design of a peptide-targeted St-PGA nanosystem. Regarding the administration route of peptide-guided St-PGA nanosystems, in the future we also wish to evaluate the intravenous route, which, barring the case of intraperitoneal chemotherapy, represents a more clinically translatable route to deliver cancer therapies.

In the OximUNO system, drug release studies revealed only 15% DOX release, which agrees with our previous studies (54, 56) but suggests room for improvement, which may come from using longer polymer-drug linkers such as EMCH (N-ε-maleimidocaproic acid hydrazide) moiety (54, 56) or from the use of external triggers (88–90). Unexpectedly, we failed to observe a significant increase in DOX release in the presence of cathepsin B with respect to the hydrolytic conditions; we hypothesize that the nanoconjugate conformation slows down proteolytic degradation, hampering *in vitro* quantification within the studied timeframe (53).

Our *in vivo* efficacy studies showed that, strikingly, the sole depletion of CD206^+^ TAMs with OximUNO alleviated tumoral immunosuppression and reduced dissemination and growth, confirming the protumoral and immunosuppressive roles assigned to CD206^+^ TAMs in the literature and reaffirming the importance of targeting this particular TAM subset. In addition, the observed reduction in the number of CD206^+^ TAMs and CD31^+^ structures for OximUNO agrees with the established angiogenic role of CD206^+^ TAMs (24).

From a safety point of view, we found that the OximUNO nanoformulation of DOX had the least negative impact on mouse bodyweight compared with free DOX or the untargeted nanoformulation St-PGA-DOX. In addition, OximUNO did not alter Crea or ALAT levels, indicating the absence of acute hepatic or renal toxicity. Our data suggest that the signal observed in the kidneys for St-PGA-OG-mUNO [consistent with the previously reported excretion of St-PGA (53, 83)] did not translate into acute renal toxicity for OximUNO. These are relevant findings as DOX induces cell death and tissue damage not only in the heart but also in the liver and kidneys (91). OximUNO did not affect or alter the macrophage populations of the spleen *in vivo*, in agreement with the absence of spleen targeting we observed for St-PGA-OG-mUNO.

Most preclinical studies evaluating the effect of M2 TAM targeted monotherapy in the 4T1 mouse model have either not shown efficacy on secondary tumors (92, 93), a lack of efficacy in primary tumors or metastases in the case of anti-CLEVER-1 (70), or a prometastatic effect in the case of anti-CSF1R (94). Hence, along with anti-MARCO therapy (95), OximUNO constitutes one of the few reports of an M2 TAM-targeted monotherapy affecting both primary and secondary tumors in the 4T1 mouse model.

Beyond TAM depletion, we show that St-PGA-mUNO represents an attractive platform to carry additional therapeutic payloads other than DOX, which could include M2→M1 polarizing agents such as TLR7 agonists (44, 96), beta-emitting radiotherapeutic agents such as dodecanetetraacetic acid–chelated ^177^Lu (97), or photosensitizers used in photodynamic therapy (88–90). We also envisage the combination of TAM depletion via OximUNO administration together with current chemotherapy regimens to prevent dissemination and relapse, or the use of OximUNO prior to surgery, that is, as neoadjuvant chemotherapy.

Taking OximUNO as a proof of concept, our data support the peptide-targeted St-PGA design reported here as a new targeted nanosystem that could target other receptors by exchanging the targeting peptide.

## Supplementary Material

Supplementary Data S1 One .docx word file containing the following information:
Scheme S1. Synthetic approach for St-PGA-OG-mUNO.
Scheme S2. Synthetic approach for OximUNO.
Video S1. Fifty ns MD trajectory of St-PGA in solution.
Table S1. St-PGA-OG-mUNO and St-PGA-OG dye loading, mUNO loading, size and charge.
Table S2. OximUNO and St-PGA-DOX drug loading, mUNO loading, size and charge.
Table S3. Hepatic and renal toxicity analysed following one dose of OximUNO.
Fig. S1. Representative characterisation of St-PGA-OG and St-PGA-OG-mUNO.
Fig. S2. The angle used to characterise the rotation of mUNO around PGA.
Fig. S3. St-PGA-OG-mUNO shows low hepatic accumulation in the orthotopic TNBC.
Fig. S4. Immunostaining showing CD206 expression in the liver compared to the saturated signal of CD206 in the orthotopic 4T1 tumour.
Fig. S5. Immunostaining of endogenous IgG indicates leaky tumour vasculature in the orthotopic 4T1 tumours.
Fig. S6. Distribution of St-PGA-OG-mUNO in the lung, spleen, sentinel lymph node (SLN) and kidney.
Fig. S7. St-PGA-OG-mUNO shows low hepatic accumulation in the experimental metastasis of TNBC.
Fig. S8. St-PGA-OG-mUNO shows higher receptor colocalisation than FAM-mUNO.
Fig. S9. St-PGA-OG-mUNO shows high homing to M2 TAMs in the orthotopic TNBC but higher hepatic accumulation with a higher dose.
Fig. S10. Representative characterisation of St-PGA-DOX and OximUNO.
Fig. S11. LC-MS method development for the determination of DOX in drug release studies and stability studies of OximUNO in i.p. fluid and dPBS.
Fig. S12. OximUNO shows no signs of hepatic or renal toxicity.
Fig. S13. H&E on healthy Balb/c mouse lung.
Fig. S14. Localisation of St-PGA-OG/St-PGA-OG-mUNO and tumour vessels.
Fig. S15. H&E staining of hearts from the treatment of orthotopic TNBC.
Fig. S16. A repetition of OximUNO treatment on the orthotopic TNBC monitoring tumour growth and survival.
Fig. S17. Flow cytometry plots for M2 and M1 macrophages.
Fig. S18. Flow cytometry gating for cytotoxic T lymphocytes (CTLs).
Fig. S19. Flow cytometry gating for T regulatory cells (Tregs).
Fig. S20. Flow cytometry analysis showing total cells.
Fig. S21. Treatment with OximUNO does not affect splenic macrophages.

## References

[1] Rivenbark AG , O'Connor SM, Coleman WB. Molecular and cellular heterogeneity in breast cancer: challenges for personalized medicine. Am J Pathol 2013;183:1113–24. 23993780 10.1016/j.ajpath.2013.08.002 PMC5691324

[2] Foulkes WD , Smith IE, Reis-Filho JS. Triple-negative breast cancer. N Engl J Med 2010;363:1938–48. 21067385 10.1056/NEJMra1001389

[3] Lehmann BD , Bauer JA, Chen X, Sanders ME, Chakravarthy AB, Shyr Y, . Identification of human triple-negative breast cancer subtypes and preclinical models for selection of targeted therapies. J Clin Invest 2011;121:2750–67. 21633166 10.1172/JCI45014 PMC3127435

[4] Garrido-Castro AC , Lin NU, Polyak K. Insights into molecular classifications of triple-negative breast cancer: improving patient selection for treatment. Cancer Discov 2019;9:176–98. 30679171 10.1158/2159-8290.CD-18-1177 PMC6387871

[5] Pardoll DM . The blockade of immune checkpoints in cancer immunotherapy. Nat Rev Cancer 2012;12:252–64. 22437870 10.1038/nrc3239 PMC4856023

[6] Esfahani K , Roudaia L, Buhlaiga N, Del Rincon SV, Papneja N, Miller WH Jr. A review of cancer immunotherapy: from the past, to the present, to the future. Curr Oncol 2020;27:S87–S97. 32368178 10.3747/co.27.5223 PMC7194005

[7] Adams S , Gatti-Mays ME, Kalinsky K, Korde LA, Sharon E, Amiri-Kordestani L, . Current landscape of immunotherapy in breast cancer: a review. JAMA Oncol 2019;5:1205–14. 30973611 10.1001/jamaoncol.2018.7147 PMC8452050

[8] Gong J , Chehrazi-Raffle A, Reddi S, Salgia R. Development of PD-1 and PD-L1 inhibitors as a form of cancer immunotherapy: a comprehensive review of registration trials and future considerations. J Immunother Cancer 2018;6:8. 29357948 10.1186/s40425-018-0316-z PMC5778665

[9] Schmid P , Adams S, Rugo HS, Schneeweiss A, Barrios CH, Iwata H, . Atezolizumab and nab-paclitaxel in advanced triple-negative breast cancer. N Engl J Med 2018;379:2108–21. 30345906 10.1056/NEJMoa1809615

[10] Schmid P , Rugo HS, Adams S, Schneeweiss A, Barrios CH, Iwata H, . Atezolizumab plus nab-paclitaxel as first-line treatment for unresectable, locally advanced or metastatic triple-negative breast cancer (IMpassion130): updated efficacy results from a randomised, double-blind, placebo-controlled, phase 3 trial. Lancet Oncol 2020;21:44–59. 31786121 10.1016/S1470-2045(19)30689-8

[11] Marra A , Viale G, Curigliano G. Recent advances in triple negative breast cancer: the immunotherapy era. BMC Med 2019;17:90. 31068190 10.1186/s12916-019-1326-5 PMC6507064

[12] Mori H , Kubo M, Yamaguchi R, Nishimura R, Osako T, Arima N, . The combination of PD-L1 expression and decreased tumor-infiltrating lymphocytes is associated with a poor prognosis in triple-negative breast cancer. Oncotarget 2017;8:15584–92. 28107186 10.18632/oncotarget.14698 PMC5362507

[13] Mittendorf EA , Philips AV, Meric-Bernstam F, Qiao N, Wu Y, Harrington S, . PD-L1 expression in triple-negative breast cancer. Cancer Immunol Res 2014;2:361–70. 24764583 10.1158/2326-6066.CIR-13-0127 PMC4000553

[14] Socinski MA , Jotte RM, Cappuzzo F, Orlandi F, Stroyakovskiy D, Nogami N, . Atezolizumab for first-line treatment of metastatic nonsquamous NSCLC. N Engl J Med 2018;378:2288–301. 29863955 10.1056/NEJMoa1716948

[15] Adams S , Diéras V, Barrios CH, Winer EP, Schneeweiss A, Iwata H, . Patient-reported outcomes from the phase III IMpassion130 trial of atezolizumab plus nab-paclitaxel in metastatic triple-negative breast cancer. Ann Oncol 2020;31:582–9. 32178964 10.1016/j.annonc.2020.02.003

[16] Fecher LA , Agarwala SS, Hodi FS, Weber JS. Ipilimumab and its toxicities: a multidisciplinary approach. Oncologist 2013;18:733–43. 23774827 10.1634/theoncologist.2012-0483 PMC4063401

[17] Hunter G , Voll C, Robinson CA. Autoimmune inflammatory myopathy after treatment with ipilimumab. Can J Neurol Sci 2009;36:518–20. 19650371 10.1017/s0317167100007939

[18] Maker AV , Phan GQ, Attia P, Yang JC, Sherry RM, Topalian SL, . Tumor regression and autoimmunity in patients treated with cytotoxic T lymphocyte–associated antigen 4 blockade and interleukin 2: a phase I/II study. Ann Surg Oncol 2005;12:1005–16. 16283570 10.1245/ASO.2005.03.536 PMC1473970

[19] Phan GQ , Yang JC, Sherry RM, Hwu P, Topalian SL, Schwartzentruber DJ, . Cancer regression and autoimmunity induced by cytotoxic T lymphocyte-associated antigen 4 blockade in patients with metastatic melanoma. Proc Natl Acad Sci U S A 2003;100:8372–7. 12826605 10.1073/pnas.1533209100 PMC166236

[20] Cretella D , Fumarola C, Bonelli M, Alfieri R, La Monica S, Digiacomo G, . Pre-treatment with the CDK4/6 inhibitor palbociclib improves the efficacy of paclitaxel in TNBC cells. Sci Rep 2019;9:13014. 31506466 10.1038/s41598-019-49484-4 PMC6736958

[21] Arola OJ , Saraste A, Pulkki K, Kallajoki M, Parvinen M, Voipio-Pulkki LM. Acute doxorubicin cardiotoxicity involves cardiomyocyte apoptosis. Cancer Res 2000;60:1789–92. 10766158

[22] Zhang S , Liu X, Bawa-Khalfe T, Lu LS, Lyu YL, Liu LF, . Identification of the molecular basis of doxorubicin-induced cardiotoxicity. Nat Med 2012;18:1639–42. 23104132 10.1038/nm.2919

[23] Keklikoglou I , Cianciaruso C, Güç E, Squadrito ML, Spring LM, Tazzyman S, . Chemotherapy elicits pro-metastatic extracellular vesicles in breast cancer models. Nat Cell Biol 2019;21:190–202. 30598531 10.1038/s41556-018-0256-3 PMC6525097

[24] Hughes R , Qian BZ, Rowan C, Muthana M, Keklikoglou I, Olson OC, . Perivascular M2 macrophages stimulate tumor relapse after chemotherapy. Cancer Res 2015;75:3479–91. 26269531 10.1158/0008-5472.CAN-14-3587 PMC5024531

[25] Lewis CE , Pollard JW. Distinct role of macrophages in different tumor microenvironments. Cancer Res 2006;66:605–12. 16423985 10.1158/0008-5472.CAN-05-4005

[26] Peranzoni E , Lemoine J, Vimeux L, Feuillet V, Barrin S, Kantari-Mimoun C, . Macrophages impede CD8 T cells from reaching tumor cells and limit the efficacy of anti–PD-1 treatment. Proc Natl Acad Sci U S A 2018;115:E4041–50. 29632196 10.1073/pnas.1720948115 PMC5924916

[27] Neubert NJ , Schmittnaegel M, Bordry N, Nassiri S, Wald N, Martignier C, . T cell–induced CSF1 promotes melanoma resistance to PD1 blockade. Sci Transl Med 2018;10:eaan3311. 29643229 10.1126/scitranslmed.aan3311 PMC5957531

[28] Daurkin I , Eruslanov E, Stoffs T, Perrin GQ, Algood C, Gilbert SM, . Tumor-associated macrophages mediate immunosuppression in the renal cancer microenvironment by activating the 15-lipoxygenase-2 pathway. Cancer Res 2011;71:6400–9. 21900394 10.1158/0008-5472.CAN-11-1261

[29] Gok Yavuz B , Gunaydin G, Gedik ME, Kosemehmetoglu K, Karakoc D, Ozgur F, . Cancer associated fibroblasts sculpt tumour microenvironment by recruiting monocytes and inducing immunosuppressive PD-1 ^+^ TAMs. Sci Rep 2019;9:3172. 30816272 10.1038/s41598-019-39553-z PMC6395633

[30] Pathria P , Louis TL, Varner JA. Targeting tumor-associated macrophages in cancer. Trends Immunol 2019;40:310–27. 30890304 10.1016/j.it.2019.02.003

[31] DeNardo DG , Brennan DJ, Rexhepaj E, Ruffell B, Shiao SL, Madden SF, . Leukocyte complexity predicts breast cancer survival and functionally regulates response to chemotherapy. Cancer Discov 2011;1:54–67. 22039576 10.1158/2159-8274.CD-10-0028 PMC3203524

[32] Mancini VSBW , Pasquini JM, Correale JD, Pasquini LA. Microglial modulation through colony-stimulating factor-1 receptor inhibition attenuates demyelination. Glia 2019;67:291–308. 30456797 10.1002/glia.23540

[33] Lee S , Shi XQ, Fan A, West B, Zhang J. Targeting macrophage and microglia activation with colony stimulating factor 1 receptor inhibitor is an effective strategy to treat injury-triggered neuropathic pain. Mol Pain 2018;14:1744806918764979. 29546785 10.1177/1744806918764979 PMC5858622

[34] Bissinger S , Hage C, Wagner V, Maser IP, Brand V, Schmittnaegel M, . Macrophage depletion induces edema through release of matrix-degrading proteases and proteoglycan deposition. Sci Transl Med 2021;13:eabd4550. 34135110 10.1126/scitranslmed.abd4550

[35] Wesolowski R , Sharma N, Reebel L, Rodal MB, Peck A, West BL, . Phase Ib study of the combination of pexidartinib (PLX3397), a CSF-1R inhibitor, and paclitaxel in patients with advanced solid tumors. Ther Adv Med Oncol 2019;11:1758835919854238. 31258629 10.1177/1758835919854238 PMC6589951

[36] Papadopoulos KP , Gluck L, Martin LP, Olszanski AJ, Tolcher AW, Ngarmchamnanrith G, . First-in-human study of AMG 820, a monoclonal anti-colony-stimulating factor 1 receptor antibody, in patients with advanced solid tumors. Clin Cancer Res 2017;23:5703–10. 28655795 10.1158/1078-0432.CCR-16-3261

[37] Kitamura T , Doughty-Shenton D, Cassetta L, Fragkogianni S, Brownlie D, Kato Y, . Monocytes differentiate to immune suppressive precursors of metastasis-associated macrophages in mouse models of metastatic breast cancer. Front Immunol 2018;8:2004. 29387063 10.3389/fimmu.2017.02004 PMC5776392

[38] Madsen DH , Jürgensen HJ, Siersbæk MS, Kuczek DE, Grey Cloud L, Liu S, . Tumor-associated macrophages derived from circulating inflammatory monocytes degrade collagen through cellular uptake. Cell Rep 2017;21:3662–71. 29281816 10.1016/j.celrep.2017.12.011 PMC5753792

[39] Ishihara D , Dovas A, Hernandez L, Pozzuto M, Wyckoff J, Segall JE, . Wiskott-aldrich syndrome protein regulates leukocyte-dependent breast cancer metastasis. Cell Rep 2013;4:429–36. 23911287 10.1016/j.celrep.2013.07.007 PMC3777703

[40] Karousou E , D'Angelo ML, Kouvidi K, Vigetti D, Viola M, Nikitovic D, . Collagen VI and hyaluronan: the common role in breast cancer. Biomed Res Int 2014;2014:606458. 25126569 10.1155/2014/606458 PMC4121998

[41] Scodeller P , Simón-Gracia L, Kopanchuk S, Tobi A, Kilk K, Säälik P, . Precision targeting of tumor macrophages with a CD206 binding peptide. Sci Rep 2017;7:14655. 29116108 10.1038/s41598-017-14709-x PMC5676682

[42] Lepland A , Asciutto EK, Malfanti A, Simón-Gracia L, Sidorenko V, Vicent MJ, . Targeting pro-tumoral macrophages in early primary and metastatic breast tumors with the CD206-binding mUNO peptide. Mol Pharm 2020;17:2518–31. 32421341 10.1021/acs.molpharmaceut.0c00226

[43] Asciutto EK , Kopanchuk S, Lepland A, Simón-Gracia L, Aleman C, Teesalu T, . Phage-display-derived peptide binds to human CD206 and modeling reveals a new binding site on the receptor. J Phys Chem B 2019;123:1973–82. 30768279 10.1021/acs.jpcb.8b11876

[44] Figueiredo P , Lepland A, Scodeller P, Fontana F, Torrieri G, Tiboni M, . Peptide-guided resiquimod-loaded lignin nanoparticles convert tumor-associated macrophages from M2 to M1 phenotype for enhanced chemotherapy. Acta Biomater 2021;133:231–43. 33011297 10.1016/j.actbio.2020.09.038

[45] Jameson B , Baribaud F, Pöhlmann S, Ghavimi D, Mortari F, Doms RW, . Expression of DC-SIGN by dendritic cells of intestinal and genital mucosae in humans and rhesus macaques. J Virol 2002;76:1866–75. 11799181 10.1128/JVI.76.4.1866-1875.2002 PMC135921

[46] Conniot J , Scomparin A, Peres C, Yeini E, Pozzi S, Matos AI, . Immunization with mannosylated nanovaccines and inhibition of the immune-suppressing microenvironment sensitizes melanoma to immune checkpoint modulators. Nat Nanotechnol 2019;14:891–901. 31384037 10.1038/s41565-019-0512-0

[47] Tian C , Kasavajhala K, Belfon KAA, Raguette L, Huang H, Migues AN, . ff19SB: amino-acid-specific protein backbone parameters trained against quantum mechanics energy surfaces in solution. J Chem Theory Comput 2020;16:528–52. 31714766 10.1021/acs.jctc.9b00591 PMC13071887

[48] Hopkins CW , Le Grand S, Walker RC, Roitberg AE. Long-time-step molecular dynamics through hydrogen mass repartitioning. J Chem Theory Comput 2015;11:1864–74. 26574392 10.1021/ct5010406

[49] Berendsen HJC , Postma JPM, van Gunsteren WF, DiNola A, Haak JR. Molecular dynamics with coupling to an external bath. J Chem Phys 1984;81:3684–90.

[50] Ryckaert JP , Ciccotti G, Berendsen HJC. Numerical integration of the cartesian equations of motion of a system with constraints: molecular dynamics of n-alkanes. J Comput Phys 1977;23:327–41.

[51] Ray A , Dittel BN. Isolation of mouse peritoneal cavity cells. J Vis Exp 2010:1488. 20110936 10.3791/1488 PMC3152216

[52] Bankhead P , Loughrey MB, Fernández JA, Dombrowski Y, McArt DG, Dunne PD, . QuPath: open source software for digital pathology image analysis. Sci Rep 2017;7:16878. 29203879 10.1038/s41598-017-17204-5 PMC5715110

[53] Duro-Castano A , England RM, Razola D, Romero E, Oteo-Vives M, Morcillo MA, . Well-defined star-shaped polyglutamates with improved pharmacokinetic profiles as excellent candidates for biomedical applications. Mol Pharm 2015;12:3639–49. 26355563 10.1021/acs.molpharmaceut.5b00358

[54] Arroyo-Crespo JJ , Armiñán A, Charbonnier D, Balzano-Nogueira L, Huertas-López F, Martí C, . Tumor microenvironment-targeted poly-L-glutamic acid-based combination conjugate for enhanced triple negative breast cancer treatment. Biomaterials 2018;186:8–21. 30278346 10.1016/j.biomaterials.2018.09.023

[55] Duro-Castano A , Sousa-Herves A, Armiñán A, Charbonnier D, Arroyo-Crespo JJ, Wedepohl S, . Polyglutamic acid-based crosslinked doxorubicin nanogels as an anti-metastatic treatment for triple negative breast cancer. J Control Release 2021;332:10–20. 33587988 10.1016/j.jconrel.2021.02.005

[56] Arroyo-Crespo JJ , Deladriere C, Nebot VJ, Charbonnier D, Masiá E, Paul A, . Anticancer activity driven by drug linker modification in a polyglutamic acid-based combination-drug conjugate. Adv Funct Mater 2018;28:1800931.

[57] Shaffer SA , Baker-Lee C, Kennedy J, Lai MS, de Vries P, Buhler K, . *In vitro* and *in vivo* metabolism of paclitaxel poliglumex: identification of metabolites and active proteases. Cancer Chemother Pharmacol 2007;59:537–48. 16924498 10.1007/s00280-006-0296-4

[58] Bertani FR , Mozetic P, Fioramonti M, Iuliani M, Ribelli G, Pantano F, . Classification of M1/M2-polarized human macrophages by label-free hyperspectral reflectance confocal microscopy and multivariate analysis. Sci Rep 2017;7:8965. 28827726 10.1038/s41598-017-08121-8 PMC5566322

[59] Gordon SR , Maute RL, Dulken BW, Hutter G, George BM, McCracken MN, . PD-1 expression by tumour-associated macrophages inhibits phagocytosis and tumour immunity. Nature 2017;545:495–9. 28514441 10.1038/nature22396 PMC5931375

[60] Zhang M , Hutter G, Kahn SA, Azad TD, Gholamin S, Xu CY, . Anti-CD47 treatment stimulates phagocytosis of glioblastoma by M1 and M2 polarized macrophages and promotes M1 polarized macrophages *in vivo*. PLoS One 2016;11:e0153550. 27092773 10.1371/journal.pone.0153550 PMC4836698

[61] Simon-Gracia L , Savier E, Parizot C, Brossas JY, Loisel S, Teesalu T, . Bifunctional therapeutic peptides for targeting malignant B cells and hepatocytes: proof of concept in chronic lymphocytic leukemia. Adv Ther 2020;3:2000131.

[62] Cassetta L , Kitamura T. Targeting tumor-associated macrophages as a potential strategy to enhance the response to immune checkpoint inhibitors. Front Cell Dev Biol 2018;6:38. 29670880 10.3389/fcell.2018.00038 PMC5893801

[63] Santoni M , Romagnoli E, Saladino T, Foghini L, Guarino S, Capponi M, . Triple negative breast cancer: key role of tumor-associated Macrophages in regulating the activity of anti-PD-1/PD-L1 agents. Biochim Biophys Acta Rev Cancer 2018;1869:78–84. 29126881 10.1016/j.bbcan.2017.10.007

[64] Rodell CB , Arlauckas SP, Cuccarese MF, Garris CS, Li R, Ahmed MS, . TLR7/8-agonist-loaded nanoparticles promote the polarization of tumour-associated macrophages to enhance cancer immunotherapy. Nat Biomed Eng 2018;2:578–88. 31015631 10.1038/s41551-018-0236-8 PMC6192054

[65] Loeuillard E , Yang J, Buckarma E, Wang J, Liu Y, Conboy C, . Targeting tumor-associated macrophages and granulocytic myeloid-derived suppressor cells augments PD-1 blockade in cholangiocarcinoma. J Clin Invest 2020;130:5380–96. 32663198 10.1172/JCI137110 PMC7524481

[66] Choo YW , Kang M, Kim HY, Han J, Kang S, Lee JR, . M1 macrophage-derived nanovesicles potentiate the anticancer efficacy of immune checkpoint inhibitors. ACS Nano 2018;12:8977–93. 30133260 10.1021/acsnano.8b02446

[67] Arlauckas SP , Garren SB, Garris CS, Kohler RH, Oh J, Pittet MJ, . Arg1 expression defines immunosuppressive subsets of tumor-associated macrophages. Theranostics 2018;8:5842–54. 30613266 10.7150/thno.26888 PMC6299430

[68] Landry AP , Balas M, Alli S, Spears J, Zador Z. Distinct regional ontogeny and activation of tumor associated macrophages in human glioblastoma. Sci Rep 2020;10:19542. 33177572 10.1038/s41598-020-76657-3 PMC7658345

[69] Zheng X , Weigert A, Reu S, Guenther S, Mansouri S, Bassaly B, . Spatial density and distribution of tumor-associated macrophages predict survival in non–small cell lung carcinoma. Cancer Res 2020;80:4414–25. 32699134 10.1158/0008-5472.CAN-20-0069

[70] Viitala M , Virtakoivu R, Tadayon S, Rannikko J, Jalkanen S, Hollmén M. Immunotherapeutic blockade of macrophage clever-1 reactivates the CD8 ^+^ T-cell response against immunosuppressive tumors. Clin Cancer Res 2019;25:3289–303. 30755440 10.1158/1078-0432.CCR-18-3016

[71] Etzerodt A , Tsalkitzi K, Maniecki M, Damsky W, Delfini M, Baudoin E, . Specific targeting of CD163 ^+^ TAMs mobilizes inflammatory monocytes and promotes T cell–mediated tumor regression. J Exp Med 2019;216:2394–411. 31375534 10.1084/jem.20182124 PMC6781002

[72] Linde N , Casanova-Acebes M, Sosa MS, Mortha A, Rahman A, Farias E, . Macrophages orchestrate breast cancer early dissemination and metastasis. Nat Commun 2018;9:21. 29295986 10.1038/s41467-017-02481-5 PMC5750231

[73] Witschen PM , Chaffee TS, Brady NJ, Huggins DN, Knutson TP, LaRue RS, . Tumor cell associated hyaluronan-CD44 signaling promotes pro-tumor inflammation in breast cancer. Cancers 2020;12:1325. 32455980 10.3390/cancers12051325 PMC7281239

[74] Guo C , Chen Y, Gao W, Chang A, Ye Y, Shen W, . Liposomal nanoparticles carrying anti-IL6R antibody to the tumour microenvironment inhibit metastasis in two molecular subtypes of breast cancer mouse models. Theranostics 2017;7:775–88. 28255366 10.7150/thno.17237 PMC5327649

[75] Movahedi K , Schoonooghe S, Laoui D, Houbracken I, Waelput W, Breckpot K, . Nanobody-based targeting of the macrophage mannose receptor for effective *in vivo* imaging of tumor-associated macrophages. Cancer Res 2012;72:4165–77. 22719068 10.1158/0008-5472.CAN-11-2994

[76] Azad AK , Rajaram MVS, Metz WL, Cope FO, Blue MS, Vera DR, . γ-tilmanocept, a new radiopharmaceutical tracer for cancer sentinel lymph nodes, binds to the mannose receptor (CD206). J Immunol 2015;195:2019–29. 26202986 10.4049/jimmunol.1402005 PMC4543904

[77] Jaynes JM , Lopez HW, Martin GR, YATES C, Garvin CE. Peptides having anti-inflammatory properties, US9492499B2; 2016.

[78] Scodeller P , Asciutto EK. Targeting tumors using peptides. Molecules 2020;25:808. 32069856 10.3390/molecules25040808 PMC7070747

[79] Ekladious I , Colson YL, Grinstaff MW. Polymer–drug conjugate therapeutics: advances, insights and prospects. Nat Rev Drug Discov 2019;18:273–94. 30542076 10.1038/s41573-018-0005-0 PMC12032968

[80] Duro-Castano A , Conejos-Sánchez I, Vicent MJ. Peptide-based polymer therapeutics. Polymers 2014;6:515–51.

[81] Moura LIF , Malfanti A, Peres C, Matos AI, Guegain E, Sainz V, . Functionalized branched polymers: promising immunomodulatory tools for the treatment of cancer and immune disorders. Mater Horiz 2019;6:1956–73.

[82] Melnyk T , Đorđević S, Conejos-Sánchez I, Vicent MJ. Therapeutic potential of polypeptide-based conjugates: rational design and analytical tools that can boost clinical translation. Adv Drug Deliv Rev 2020;160:136–69. 33091502 10.1016/j.addr.2020.10.007

[83] Duro-Castano A , Nebot VJ, Niño-Pariente A, Armiñán A, Arroyo-Crespo JJ, Paul A, . Capturing “extraordinary” soft-assembled charge-like polypeptides as a strategy for nanocarrier design. Adv Mater 2017;29. 10.1002/adma.201702888 28834624

[84] Duro-Castano A , Movellan J, Vicent MJ. Smart branched polymer drug conjugates as nano-sized drug delivery systems. Biomater Sci 2015;3:1321–34. 26266272 10.1039/c5bm00166h

[85] Cortez-Retamozo V , Etzrodt M, Newton A, Rauch PJ, Chudnovskiy A, Berger C, . Origins of tumor-associated macrophages and neutrophils. Proc Natl Acad Sci U S A 2012;109:2491–6. 22308361 10.1073/pnas.1113744109 PMC3289379

[86] Kurashige M , Kohara M, Ohshima K, Tahara S, Hori Y, Nojima S, . Origin of cancer-associated fibroblasts and tumor-associated macrophages in humans after sex-mismatched bone marrow transplantation. Commun Biol 2018;1:131. 30272010 10.1038/s42003-018-0137-0 PMC6123637

[87] Veglia F , Gabrilovich DI. Dendritic cells in cancer: the role revisited. Curr Opin Immunol 2017;45:43–51. 28192720 10.1016/j.coi.2017.01.002 PMC5449252

[88] Agostinis P , Berg K, Cengel KA, Foster TH, Girotti AW, Gollnick SO, . Photodynamic therapy of cancer: an update. CA Cancer J Clin 2011;61:250–81. 21617154 10.3322/caac.20114 PMC3209659

[89] Cheah HY , Gallon E, Dumoulin F, Hoe SZ, Japundžić-Žigon N, Glumac S, . Near-infrared activatable phthalocyanine–poly-L-glutamic acid conjugate: enhanced *in vivo* safety and antitumor efficacy toward an effective photodynamic cancer therapy. Mol Pharm 2018;15:2594–605. 29763568 10.1021/acs.molpharmaceut.8b00132

[90] Nguyen VN , Yan Y, Zhao J, Yoon J. Heavy-atom-free photosensitizers: from molecular design to applications in the photodynamic therapy of cancer. Acc Chem Res 2021;54:207–20. 33289536 10.1021/acs.accounts.0c00606

[91] Tacar O , Sriamornsak P, Dass CR. Doxorubicin: an update on anticancer molecular action, toxicity and novel drug delivery systems. J Pharm Pharmacol 2013;65:157–70. 23278683 10.1111/j.2042-7158.2012.01567.x

[92] Shan H , Dou W, Zhang Y, Qi M. Targeted ferritin nanoparticle encapsulating CpG oligodeoxynucleotides induces tumor-associated macrophage M2 phenotype polarization into M1 phenotype and inhibits tumor growth. Nanoscale 2020;12:22268–80. 33146206 10.1039/d0nr04520a

[93] Ramesh A , Brouillard A, Kumar S, Nandi D, Kulkarni A. Dual inhibition of CSF1R and MAPK pathways using supramolecular nanoparticles enhances macrophage immunotherapy. Biomaterials 2020;227:119559. 31670078 10.1016/j.biomaterials.2019.119559 PMC7238715

[94] Hollmén M , Karaman S, Schwager S, Lisibach A, Christiansen AJ, Maksimow M, . G-CSF regulates macrophage phenotype and associates with poor overall survival in human triple-negative breast cancer. OncoImmunology 2015;5:e1115177. 27141367 10.1080/2162402X.2015.1115177 PMC4839343

[95] Georgoudaki AM , Prokopec KE, Boura VF, Hellqvist E, Sohn S, Östling J, . Reprogramming tumor-associated macrophages by antibody targeting inhibits cancer progression and metastasis. Cell Rep 2016;15:2000–11. 27210762 10.1016/j.celrep.2016.04.084

[96] Zhang F , Ayaub EA, Wang B, Puchulu-Campanella E, Li YH, Hettiarachchi SU, . Reprogramming of profibrotic macrophages for treatment of bleomycin-induced pulmonary fibrosis. EMBO Mol Med 2020;12:e12034. 32597014 10.15252/emmm.202012034 PMC7411553

[97] Sartor O , de Bono J, Chi KN, Fizazi K, Herrmann K, Rahbar K, . Lutetium-177–PSMA-617 for metastatic castration-resistant prostate cancer. N Engl J Med 2021;385:1091–1103. 34161051 10.1056/NEJMoa2107322 PMC8446332

